# A meta-analysis of epigenome-wide association studies on pregnancy vitamin B12 concentrations and offspring DNA methylation

**DOI:** 10.1080/15592294.2023.2202835

**Published:** 2023-04-24

**Authors:** Giulietta S. Monasso, Thanh T. Hoang, Giulia Mancano, Sílvia Fernández-Barrés, John Dou, Vincent W.V. Jaddoe, Christian M. Page, Laura Johnson, Mariona Bustamante, Kelly M. Bakulski, Siri E. Håberg, Per M. Ueland, Thomas Battram, Simon K. Merid, Erik Melén, Doretta Caramaschi, Leanne K. Küpers, Jordi Sunyer, Wenche Nystad, Sandra G. Heil, Rebecca J. Schmidt, Martine Vrijheid, Gemma C. Sharp, Stephanie J. London, Janine F. Felix

**Affiliations:** a The Generation R Study Group, Erasmus MC, University Medical Center Rotterdam, Rotterdam, the Netherlands; b Department of Pediatrics, Erasmus MC, University Medical Center Rotterdam, Rotterdam, the Netherlands; c Epidemiology Branch, National Institute of Environmental Health Sciences, National Institutes of Health, Department of Health and Human Services, Research Triangle Park, Durham, NC, USA; d Medical Research Council Integrative Epidemiology Unit, University of Bristol, Bristol, UK; e Bristol Medical School Population Health Sciences, University of Bristol, Bristol, UK; f ISGlobal, Bacelona Institute for Global Health, Barcelona, Spain; g Universitat Pompeu Fabra (UPF), Barcelona, Spain; h CIBER Epidemiología y Salud Pública (CIBERESP), madrid,Barcelona, Spain; i Department of Epidemiology, School of Public Health, University of Michigan, Ann Arbor, USA; j Centre for Fertility and Health, Norwegian Institute of Public Health, Oslo, Norway; k Department of Mathematics, Faculty of Mathematics and Natural Sciences, University of Oslo, Oslo, Norway; l Centre for Exercise, Nutrition and Health Sciences, School for Policy Studies, University of Bristol, Bristol, UK; m BEVITAL, Bergen, Norway; n Institute of Environmental Medicine, Karolinska Institutet, Stockholm, Sweden; o Department of Clinical Sciences and Education, Södersjukhuset, Karolinska Institutet, Stockholm, Sweden; p Sachs’ Children’s Hospital, South General Hospital, Stockholm, Sweden; q College of Life and Environmental Sciences, Department of Psychology, University of Exeter, Exeter, UK; r Division of Human Nutrition and Health, Wageningen University, Wageningen, The Netherlands; s IMIM (Hospital del Mar Medical Research Institute), Barcelona, Spain; t Department of Chronic Diseases and Ageing, Norwegian Institute of Public Health, Oslo, Norway; u Department of Clinical Chemistry, Erasmus MC, University Medical Center Rotterdam, Rotterdam, the Netherlands; v Department of Public Health Sciences, School of Medicine, University of California Davis, Davis, USA; w The UC Davis MIND Institute, School of Medicine, University of California Davis, Sacramento, USA

**Keywords:** Vitamin B12, DNA methylation, epidemiology, cohort study, meta-analysis, PACE consortium

## Abstract

Circulating vitamin B12 concentrations during pregnancy are associated with offspring health. Foetal DNA methylation changes could underlie these associations. Within the Pregnancy And Childhood Epigenetics Consortium, we meta-analysed epigenome-wide associations of circulating vitamin B12 concentrations in mothers during pregnancy (*n* = 2,420) or cord blood (*n* = 1,029), with cord blood DNA methylation. Maternal and newborn vitamin B12 concentrations were associated with DNA methylation at 109 and 7 CpGs, respectively (False Discovery Rate *P*-value <0.05). Persistent associations with DNA methylation in the peripheral blood of up to 482 children aged 4–10 y were observed for 40.7% of CpGs associated with maternal vitamin B12 and 57.1% of CpGs associated with newborn vitamin B12. Of the CpGs identified in the maternal meta-analyses, 4.6% were associated with either birth weight or gestational age in a previous work. For the newborn meta-analysis, this was the case for 14.3% of the identified CpGs. Also, of the CpGs identified in the newborn meta-analysis, 14.3% and 28.6%, respectively, were associated with childhood cognitive skills and nonverbal IQ. Of the 109 CpGs associated with maternal vitamin B12, 18.3% were associated with nearby gene expression. In this study, we showed that maternal and newborn vitamin B12 concentrations are associated with DNA methylation at multiple CpGs in offspring blood (*P*_FDR_<0.05). Whether this differential DNA methylation underlies associations of vitamin B12 concentrations with child health outcomes, such as birth weight, gestational age, and childhood cognition, should be further examined in future studies.

## Introduction

Vitamin B12 (cobalamin) is an essential nutrient for humans and can be obtained from meat, fish, dairy, eggs, and liver [1]. Lower maternal circulating vitamin B12 concentrations during pregnancy have been associated with adverse health outcomes in the offspring, including higher risk of low birth weight and preterm birth, suboptimal cardiometabolic outcomes, and lower kidney function [2–7]. Vitamin B12 concentrations typically decline during pregnancy, but a clinical cut-off for deficiency in pregnancy has not been established [8,9]. Vitamin B12 is a crucial cofactor in one-carbon metabolism. It interacts closely with folate to guarantee the availability of methyl groups by remethylating homocysteine. Methyl groups are essential for cellular growth and differentiation, nucleic acid synthesis, and DNA methylation [8]. As such, DNA methylation may represent a mechanism underlying the associations of circulating vitamin B12 concentrations during pregnancy with child health [8]. Previously, circulating vitamin B12 concentrations during pregnancy have been associated with both global and gene-specific cord blood DNA methylations in two studies among 430 and 99 newborns, respectively [10,11]. Also, a Mendelian randomization study suggested a causal role for DNA methylation in the association of maternal circulating vitamin B12 concentrations during pregnancy with child IQ [12]. Whereas a meta-analysis of two epigenome-wide association studies (EWASs) reported associations of circulating folate concentrations during pregnancy with cord blood DNA methylation at 443 cytosine-phosphate-guanine sites (CpGs), a similar large-scale EWAS on circulating vitamin B12 concentrations has not been conducted [13].

Therefore, in the Pregnancy And Childhood Epigenetics (PACE) Consortium, we meta-analysed data from four cohorts on the associations of maternal circulating vitamin B12 concentrations during pregnancy with epigenome-wide cord blood DNA methylation (‘maternal meta-analysis’) [14]. Similarly, using data from two PACE cohorts, we meta-analysed associations of cord blood vitamin B12 concentrations with epigenome-wide cord blood DNA methylation (‘newborn meta-analysis’).

## Materials and methods

We aimed to analyse associations of maternal and cord blood vitamin B12 concentrations with cord blood DNA methylation, their persistence into childhood, and their associations with child health outcomes.

### Study population

Six birth cohorts contributed to the analyses (Table 1): the Avon Longitudinal Study of Parents and Children (ALSPAC), the Generation R Study (GENR), the Sabadell subcohort of the INfancia y Medio Ambiente (INMA) Project, the Markers of Autism Risk Learning Early Signs (MARBLES), and two datasets of the Norwegian Mother Father and Child Cohort Study (MoBa1 and MoBa2) [15–21]. **Supplementary Methods** provides detailed information on the study populations. All studies were approved by the local Medical Ethical Committees, and informed consent was obtained for all participants.

**Table 1.** Subject characteristics.

	Cohort	N participants	Ancestry	Vitamin B12 concentration (pmol/L), median (95% range)	Gestational age at vitamin B12 sampling (weeks), mean (SD)	Age at DNA methylation measurement^1^, mean (SD)
**Maternal vitamin B12 concentrations**						
Meta-analysis	Total	2,420				
	GENR	823	European	180.0 (84.0, 425.2)	13.1 (1.7)	40.2 (1.5)
	INMA	372	European	222.5 (138.0, 359.8)	13.5 (1.8)	39.8 (1.4)
	MoBa1	1,007	European	297.3 (150.3, 535.9)	NA^2^	39.5 (1.6)
	MoBa2	218	European	294.4 (155.7, 529.5)	NA^2^	39.4 (1.6)
Look-up multi-ethnic population	MARBLES	48	Mixed	236.3 (150.8, 438.0)	23.0 (9.4)	39.1 (1.2)
Look-up early childhood (4–7 y)	Total	479				
	GENR	284	European	185.5 (84.3, 435.4)	40.2 (1.5)	6.0 (0.4)
	INMA	195	European	224.3 (126.5, 351.8)	39.7 (1.5)	4.5 (0.2)
Look-up late childhood (8–10 y)	Total	482				
	GENR	267	European	182.0 (78.0, 434.9)	40.1 (1.5)	9.8 (0.3)
	INMA	215	European	223.6 (139.0, 360.1)	39.9 (1.4)	8.8 (0.6)
**Newborn vitamin B12 concentrations**						
Meta-analysis	Total	1,029				
	ALSPAC	81	European	300.0 (120.0, 670.0)	40.2 (1.5)	40.2 (1.5)
	GENR	948	European	306.5 (130.2, 818.1)	39.6 (1.3)	39.6 (1.3)
Look-up early childhood (4–7 y)	Total	417				
	ALSPAC	85	European	306.0 (121.3, 665.3)	39.7 (1.2)	7.4 (0.1)
	GENR	332	European	315.5 (135.2, 861.5)	40.2 (1.5)	6.0 (0.4)
Look-up late childhood (8–10 y)	GENR	321	European	302.0 (128.2, 869.9)	40.1 (1.5)	9.8 (0.3)
Look-up adolescence (17 y)	ALSPAC	83	European	306 (120.7, 667.7)	39.6 (1.3)	17.0 (1.0)

Abbreviations: ALSPAC, Avon Longitudinal Study of Parents and Children; GENR, Generation R Study; INMA, Sabadell subcohort of the INfancia y Medio Ambiente (INMA) Project; MARBLES, Markers of Autism Risk Learning Early Signs; MoBa, Norwegian Mother, Father, and Child Cohort Study.

For analyses at birth in weeks gestational age; for analyses in childhood in years.

For MoBa1 and MoBa2, individual data on gestational age at blood sampling (study population median 18, 25th–75th percentile 16–21) weeks were not available. Gestational age at blood sampling was not included as covariate in the models.

**Figure 1.**
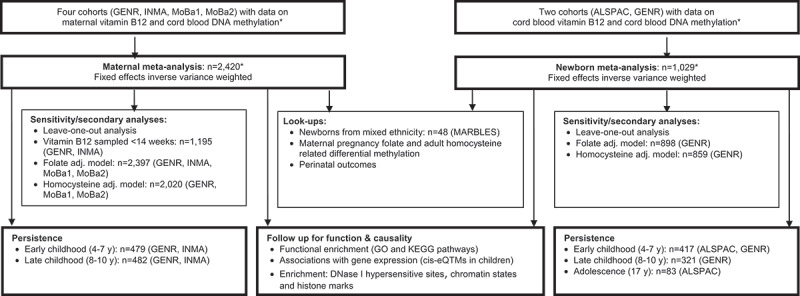
Study design. Schematic representation of the analyses of circulating vitamin B12 concentrations during foetal development and epigenome-wide DNA methylation in cord blood.

Abbreviations: adj: adjusted; *cis*-eQTM: cis-expression quantitative trait methylation; EWAS, epigenome-wide association study

Figure 1 shows a schematic overview of the study design. We conducted two meta-analyses of EWASs of vitamin B12 concentrations during foetal development, assessed either in the mothers’ peripheral blood during pregnancy or in newborns’ cord blood, with cord blood DNA methylation. All included newborns had cord blood DNA methylation available and complete information on either maternal or newborn circulating vitamin B12 concentrations and all covariates (complete case analysis). Only GENR had vitamin B12 concentrations available in both mothers and newborns. All cohorts excluded participants with circulating vitamin B12 concentrations outside ± 5 standard deviations (SD) from the mean of their study population to avoid undue influence of a very low number of extreme values on the identified population-level associations. This excluded 5 and 6 mother–newborn pairs from the maternal and newborn analysis, respectively, in the Generation R Study and four mother-newborn pairs from MoBa1. No participants from ALSPAC, MoBa2, INMA, or MARBLES were excluded because of outlying values. In addition, all twins, and in case of non-twin siblings, one child was included by selecting on completeness of the data or, if equal, randomly. We also performed sensitivity analyses, look-up analyses, and follow-up analyses using repeated blood DNA methylation data at older ages (5–17 y).

* In this complete-case analysis, cohorts excluded participants with circulating vitamin B12 concentrations outside ± 5 standard deviations (SD) from the mean this complete-case analysis, cohorts excluded participants with circulating vitamin B12 concentrations outside ± 5 standard deviations (SD) from the mean of their study population, all twins, and in case of non-twin siblings, one child was excluded by selecting on completeness of data or, if equal, randomly. We prioritized CpGs with FDR-*P*-value <0.05 and showed low heterogeneity (*I*^2^<50%) for follow-up analyses. Vitamin B12, folate, and homocysteine were measured in either serum or in plasma.

### Vitamin B12 measurements

Cohort-specific descriptions of blood sample collection, transport, storage, and analysis are described in **Supplementary Methods**. Maternal vitamin B12 concentrations were measured in venous plasma or serum, and cord blood samples were venous in GENR (for ALSPAC, it was not known whether cord blood samples were venous or arterial). Gestational age at maternal blood sampling differed between the included cohorts (Table 1).

### DNA methylation

Cohorts extracted DNA from cord blood samples, which were taken after delivery and subsequently stored. DNA was bisulphite converted using the EZ-96 DNA Methylation kit (Shallow) (Zymo Research Corporation, Irvine, USA). Samples were processed with the Illumina Infinium HumanMethylation450 or EPIC BeadChip assay. Quality control and normalization were performed independently by the individual cohorts, using their preferred method (see **Supplementary Methods** for details). Untransformed beta-values were used as the outcome measure. Outliers in methylation values, defined as values below the 25^th^ percentile minus 3 * interquartile range or above the 75^th^ percentile plus 3 * interquartile range, were excluded (Tukey method) [22]. CpGs located on the sex chromosomes were removed.

### Covariates

Cohort-specific characteristics are shown in **Supplementary Data 1–3**. All analyses were adjusted for maternal confounders (age, education, cohort definition), pre-pregnancy body mass index, smoking during pregnancy (no or firsttrimester only smoking versus sustained smoking), parity (nulliparous versus multiparous), child sex, batch (cohort-specific), and cell-type proportion (CD8+ T-cells, CD4+ T-cells, natural killer cells, B cells, monocytes, granulocytes, and nucleated red blood cells), estimated using the Bioconductor package ‘FlowSorted.CordBlood.Combined.450K’ [23]. Maternal vitamin B12 analyses were additionally adjusted for gestational age at blood sampling, as physiologically vitamin B12 concentrations decline during pregnancy [8,9]. Newborn vitamin B12 analyses were not adjusted for gestational age at birth, as we considered this to be a potential mediator [4].

### Statistical analyses

All analyses were described in a pre-specified analysis plan. Cohorts used a common script to perform independent epigenome-wide robust linear regression analyses in R 3.6.1 to assess associations of maternal or newborn circulating vitamin B12 concentrations (continuously, per SD increase) with cord blood DNA methylation, adjusting for covariates [24].

### Meta-analyses

To minimize the chance of human error, analysts from two cohorts independently performed fixed-effects inverse-variance weighted (IWV) meta-analyses using *METAL* and compared results [25]. We removed CpGs that were available in one cohort only and 44,960 cross-reactive CpGs [26,27]. In the result files of both meta-analyses, we flagged probes that map to DNA containing a single nucleotide polymorphism (SNP), to repetitive sequence elements, or to DNA harbouring an INDEL [26,27]. The final datasets included 429,952 (maternal meta-analysis) and 415,481 (newborn meta-analysis) CpGs. We accounted for multiple testing by controlling the FDR at 5%, implementing the method of Benjamini and Hochberg [28]. CpGs that were associated after applying the more stringent Bonferroni correction (two-sided *P*-value <1.2 × 10^−7^) were also noted. We annotated the nearest gene for all CpG based on the UCSC Genome Browser build GRCh37/hg19 as provided in the Illumina annotation file [29]. For both meta-analyses, we assessed inter-study heterogeneity. *A priori*, we decided that only CpGs with P_FDR_<0.05 that showed no major evidence of inter-study heterogeneity, as reflected by an I^2^ value <50%, would be taken forward for follow-up analyses. We call these ‘prioritized’ CpGs.

### Sensitivity analyses

We performed some sensitivity analyses (Figure 1). First, we ran a leave-one-out analysis for the prioritized CpGs of both meta-analyses, in which we re-ran the meta-analysis repeatedly with one of the contributing studies removed each time. We constructed forest plots to visualize the results for each CpG. Second, we re-ran the maternal meta-analysis restricted to cohorts with maternal vitamin B12 sampled in early pregnancy (<14 weeks gestational age), likely showing more comparable vitamin B12 concentrations [8,9]. We calculated Pearson’s correlation between effect estimates of the primary model versus the early-pregnancy model and examined the consistency in the direction of associations. For the prioritized CpGs of the maternal meta-analysis, we tested for interactions between circulating vitamin B12 concentrations and newborn rs3742801 ATP binding cassette subfamily D member 4 (*ABCD4*) genotype, by meta-analysing data from GENR, MoBa1, and MoBa2. *ABCD4* may be biologically relevant for foetal circulating vitamin B12 concentrations as it is involved in the intracellular transport of vitamin B12 [30]. It has been associated with adult circulating vitamin B12 concentrations in a large genome-wide association study [31].

### Secondary analyses

Among cohorts with these data available (Figure 1), we additionally adjusted the analyses for either circulating folate or homocysteine concentrations measured concurrently with vitamin B12, to examine whether this potential confounder and mediator, respectively, explained any findings. We calculated Pearson’s correlation between effect estimates of the primary models versus these secondary models and examined the consistency in the direction of associations.

### Look-up analyses

We performed several look-ups in the results of related analyses (Figure 1). First, we examined the prioritized CpGs of the maternal meta-analysis in newborn meta-analyses and vice versa. Second, we examined whether the prioritized CpGs from the maternal meta-analysis showed similar associations in a smaller genetic multi-ethnic population from the MARBLES study after adjusting for ancestry principal components [18]. Third, we explored persistence of differential methylation at birth (Figure 1). We meta-analysed whether the prioritized CpGs from the maternal meta-analysis with cord blood DNA methylation were also differentially methylated if measured in peripheral blood sampled in both early (4–7 y) and late (9–10 y) childhood (GENR and INMA). Similarly, we meta-analysed whether the prioritized CpGs from the newborn meta-analysis were also differently methylated if measured in peripheral blood DNA methylation data sampled in early childhood (ALSPAC and GENR), late childhood (GENR), and adolescence (17 y, ALSPAC). Childhood models were additionally adjusted for childhood at blood sampling. The ‘Houseman’ blood reference set was used for cell-type estimation (CD8+ T-cells, CD4+ T-cells, natural killer cells, B-cells, monocytes, and granulocytes) [32].

### Comparison with previous findings

First, for both meta-analyses, we examined whether there was enrichment among the prioritized CpGs and, using a less stringent cut-off, among CpGs with uncorrected *P*-values <0.05 and I^2^<50%, for CpGs previously identified at FDR-significance in two large meta-analyses (*n* ≈ 2000) of EWASs on circulating concentrations of either maternal folate during pregnancy or adult homocysteine [13,33]. Enrichment was calculated using a hypergeometric test in the phyper function in the R Stats package [24]. For vitamin B12, previous studies assessed vitamin B12 intake or supplementation rather than concentrations with gene-specific or global DNA methylation, and as such, we did not perform a similar analysis with results from those previous studies [34]. We did perform a look-up of the three CpGs in cord blood that were previously associated with maternal vitamin B12 concentrations as proxied by maternal fucosyltransferase 2 (*FUT2*-) genotype [12]. We also explored whether the prioritized CpGs from both meta-analyses were differentially methylated in previous EWASs of birth weight, gestational age, childhood overall cognitive skills, and childhood nonverbal IQ [35–37].

**Follow-up analyses of the identified CpG sites** To better understand potential mechanisms linking vitamin B12 and DNA methylation, we examined in Gene Ontology (GO) and Kyoto Encyclopedia of Genes and Genomes (KEGG) enrichment analyses potential functions of the prioritized CpGs (release December 2020 (GO) and release 97.0 (KEGG)). Analyses were conducted on the missMethyl R package version 1.25.0, which allows to correct for the number of probes per gene on the 450k array [38]. We also explored associations with gene expression, by comparing the prioritized CpGs with a catalogue containing 39,749 blood autosomal expression quantitative trait methylation sites (*cis*-eQTMs, 1 Mb window centred at the transcription start site). These were identified using data from 823 children of European ancestry aged 6–11 y from the Human Early-Life Exposome (HELIX) project after adjustment for sex, age, cohort, cell types, and correcting for multiple testing and are available at https://helixomics.isglobal.org/ [39]. We explored tissue or cell-type-specific signals by examining whether the prioritized CpGs of both meta-analyses were enriched in DNase I hypersensitive sites, chromatin states, and histone marks, using eFORGE v2.0. with its default settings, using data from either Consolidated Roadmap Epigenomics, ENCODE, or Blueprint [40]. We also examined the enrichment of specific transcription factor motifs using eFORGE TF [40].

## Results

### Study characteristics

We included 2,420 mother-newborn pairs of European ancestry in the maternal meta-analysis (Table 1). Maternal pregnancy circulating vitamin B12 concentrations were measured between 13 and 18 weeks gestational age and differed moderately between contributing cohorts (Table 1). We included 1,029 newborns of European ancestry in the newborn meta-analysis (Table 1). Cord blood vitamin B12 concentrations were comparable between the two contributing cohorts (Table 1) [21]. Across cohorts with these data, the correlations of maternal vitamin B12 with folate (*r* < 0.15) and homocysteine (*r* < −0.26) were low **(Supplementary Data 4)**. The same was observed in newborns from GENR. Maternal and newborn vitamin B12 correlated moderately (*r* = 0.45) in GENR.

Two cohorts with maternal vitamin B12 concentrations and both cohorts with newborn vitamin B12 concentrations had repeated blood DNA methylation data for a subgroup of the children at older ages (4–17 y) and contributed to look-up analyses (Figure 1 and Table 1).

### Maternal meta-analysis

**Figure 2.**
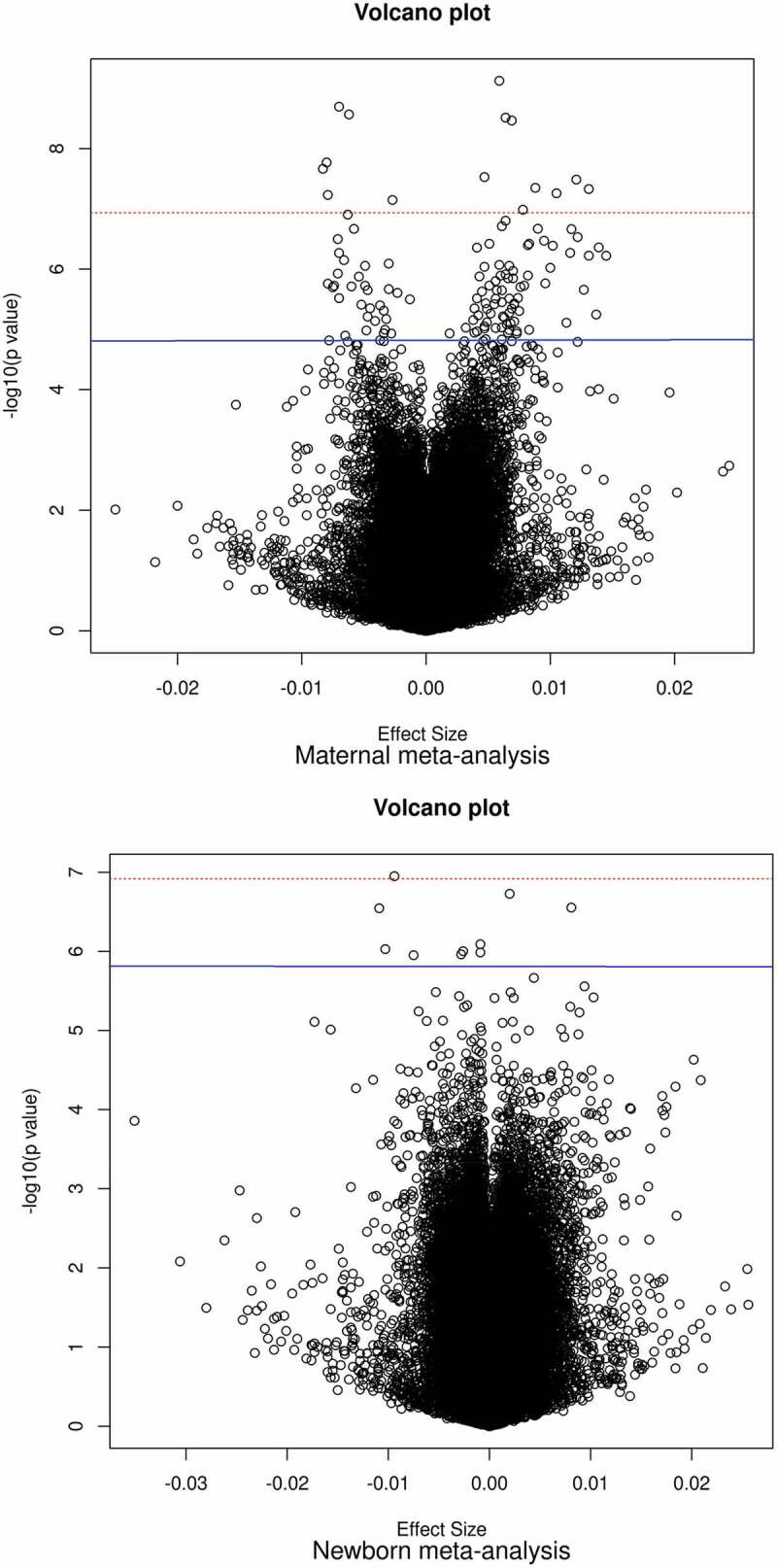
Volcano plots show the directions of associations in epigenome-wide meta-analyses of circulating vitamin B12 concentrations during foetal development.

Abbreviations: B12, vitamin B12; CpG, cytosine-phosphate-guanine site; FDR, false discovery rate; SDS, standard deviation score.

**Figure 3.**
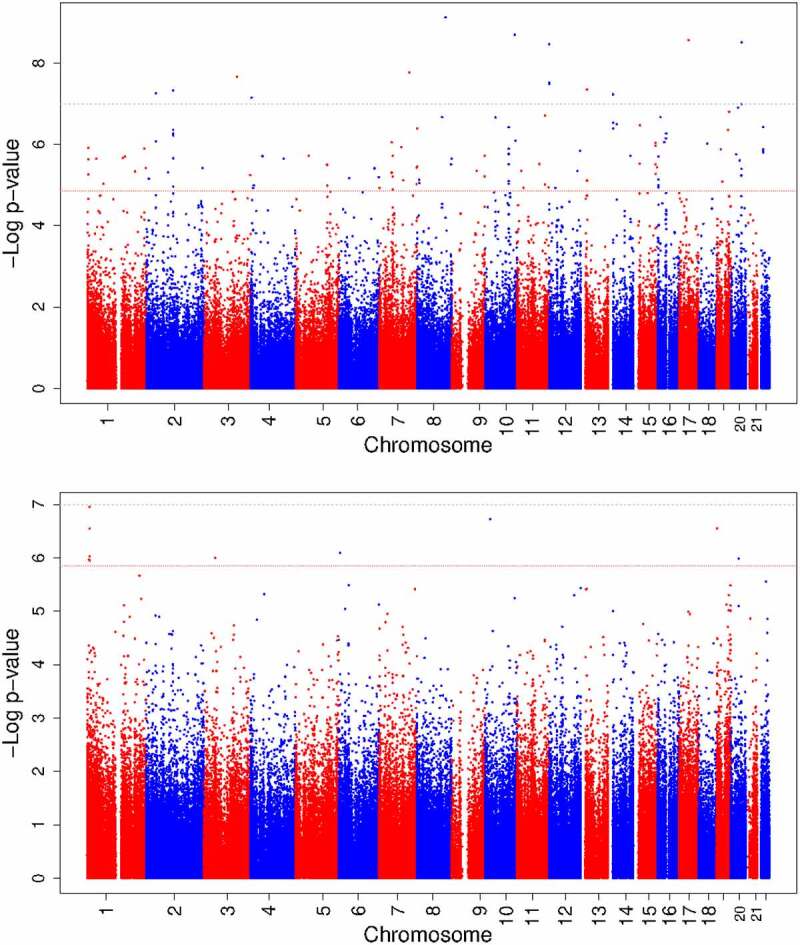
Manhattan plots of epigenome-wide meta-analyses of circulating vitamin B12 concentrations during foetal development.

Abbreviations: B12, vitamin B12; CpG, cytosine-phosphate-guanine site; FDR, false discovery rate.

Maternal vitamin B12 concentrations were associated with differential DNA methylation at 119 CpGs (*P*-value False Discovery Rate (*P*_FDR_) <0.05) in offspring cord blood after adjusting for maternal age, education, pre-pregnancy body mass index, smoking during pregnancy, parity, child sex, batch, cell-type proportions, and gestational age at blood sampling (Figures 2–3). Among all CpGs, similar numbers of positive and negative associations were observed (Figure 2). The association with the lowest *P*-value (7.49 × 10^−10^) was observed for cg25327343 (*Mal, T Cell Differentiation Protein 2* gene (*MAL2;* MIM:609684)). Per standard deviation score (SDS) (weighted mean 88.8 pmol/L) increase in vitamin B12 concentrations, DNA methylation at this CpG increased 0.60% (standard error (SE) 0.10%). Cg12889195 (*Paired box 8* gene (*PAX8*, MIM:167415)) had the largest effect size (increase in DNA methylation per SDS vitamin B12: 1.45%; SE: 0.29%; *P*-value: 6.02 × 10^−7^). We observed little evidence of inter-study heterogeneity as 109/119 (92%) CpGs had I^2^<50% (Table 2). We prioritized these 109 CpGs for follow-up analyses. **Supplementary Figure S1** shows the QQ plot of the maternal meta-analysis. There was no evidence of genomic inflation (lambda, λ = 0.98). **Supplementary Data** 5 shows the lambdas of all cohort-specific analyses and meta-analyses.

**Upper panel**: maternal meta-analysis;**Lower panel**: newborn meta-analysis

The X-axis represents the difference in DNA methylation per SDS increase in circulating vitamin B12 concentrations; the Y-axis represents the −log_10_(P). The red dotted line and the blue line represent the thresholds below which we considered associations significant using a Bonferroni correction (absolute *P*-value <1.2 × 10^−7^) and FDR-*P*-value <0.05, respectively, to account for multiple testing. In total, 56.1% of all analysed CpGs in the maternal meta-analysis showed increased methylation in relation to maternal vitamin B12 concentrations during pregnancy. Similarly, 51.4% of all analysed CpGs in the newborn meta-analysis showed increased methylation in relation to higher newborn circulating vitamin B12 concentrations.

**Upper panel**: maternal meta-analysis;**Lower panel**: newborn meta-analysis

The X-axis represents chromosomes; the Y-axis represents the −log_10_(P). The black dotted line and the red dashed line represent the thresholds below which we considered associations significant using a Bonferroni correction (absolute *P*-value <1.2 × 10^−7^) and FDR-*P*-value <0.05, respectively, to account for multiple testing. Models were adjusted for maternal confounders during pregnancy (age, education, body mass index, smoking, parity, and gestational age at blood sampling in maternal meta-analysis), child sex, cell-type proportion, and batch.

**Table 2.** Prioritized CpGs (*n* = 109) with differential methylation in cord blood in relation to maternal circulating vitamin B12 concentrations during pregnancy^1^.

CpG	Chromosome	Gene (UCSC RefGene)	Gene location (UCSC RefGene)	Flagged	Coefficient	Standard error	*P*-value	FDR-corrected *P*-value	I^2^	Persistence childhood	Overlap with folate hits in previous study^5^	Overlap with birth weight hits in previous study ^7^	Overlap with gestational age hits in previous study ^9^	Overlap with overall cognitive skills hits in previous study ^9^	Overlap with nonverbal IQ hits in previous study ^9^	eQTM	Transcript Cluster Gene
cg25327343	8	*MAL2*	Body	N	0.59	0.10	7.49 × 10^−10^	2.94 × 10^−4^	11.3	Y	N	N	N	N	N	N	
cg05665581	10	*FAM24A*	TSS1500	Y	−0.7	0.12	2.02 × 10^−9^	2.94 × 10^−4^	0.0	Y	Y	Y	N	N	N	N	
cg17900015	17	*KRT28*	TSS1500	N	−0.62	0.10	2.71 × 10^−9^	2.94 × 10^−4^	33.5	Y	Y	N	N	N	N	N	
cg00200803	20	*CDH22*	Body	Y	0.64	0.11	3.07 × 10^−9^	2.94 × 10^−4^	0.0	Y	N	N	N	N	N	N	
cg00292513	12	*IQSEC3*		N	0.69	0.12	3.42 × 10^−9^	2.94 × 10^−4^	0.0	Y^3^	N	N	N	N	N	Y	*IQSEC3*
cg15908975	7	*MIR592*		N	−0.8	0.14	1.70 × 10^−8^	1.22 × 10^−3^	0.0	Y	Y	Y	N	N	N	N	
cg08849628	3			N	−0.83	0.15	2.17 × 10^−8^	1.33 × 10^−3^	0.0	N	N	N	N	N	N	N	
cg25396728	12	*IQSEC3*		N	1.21	0.22	3.27 × 10^−8^	1.56 × 10^−3^	0.0	Y	N	N	N	N	N	Y	*IQSEC3*
cg21341928	13	*PABPC3*		N	0.88	0.16	4.49 × 10^−8^	1.83 × 10^−3^	0.0	Y	N	N	N	N	N	N	
cg19083407	2	*PAX8*		N	1.31	0.24	4.69 × 10^−8^	1.83 × 10^−3^	0.0	N	N	N	N	N	N	Y	*PAX8*
cg12302982	2			N	1.05	0.19	5.52 × 10^−8^	1.94 × 10^−3^	0.0	N	Y	N	N	N	N	N	
cg05282518	14	*OR4K2*	1stExon	N	−0.79	0.14	5.88 × 10^−8^	1.94 × 10^−3^	0.0	N	Y	N	N	N	N	N	
cg09367432	4			N	−0.27	0.05	7.13 × 10^−8^	2.19 × 10^−3^	0.0	N	N	N	N	N	N	N	
cg07296387	20	*CDH22*	Body	N	0.78	0.15	1.04 × 10^−7^	2.97 × 10^−3^	0.0	N	N	N	N	N	N	N	
cg14776195	20	*DEFB118*	TSS1500	Y	−0.63	0.12	1.25 × 10^−7^	3.36 × 10^−3^	14.4	Y^3^	N	N	N	N	N	Y	*FRG1BP*
cg19149785	19	*KLK9*		N	0.64	0.12	1.58 × 10^−7^	3.99 × 10^−3^	0.0	Y^3^	N	N	N	N	N	N	
cg06468072	11	*DSCAML1*	Body	N	0.61	0.12	1.94 × 10^−7^	4.45 × 10^−3^	0.0	N	N	N	N	N	N	N	
cg11421509	8			Y	0.9	0.17	2.14 × 10^−7^	4.45 × 10^−3^	0.0	Y^3^	N	N	N	N	N	N	
cg03873392	16			Y	−0.58	0.11	2.15 × 10^−7^	4.45 × 10^−3^	0.0	Y	N	N	N	N	N	N	
cg03327325	10			Y	1.17	0.22	2.17 × 10^−7^	4.45 × 10^−3^	4.7	Y	N	N	N	N	N	N	
cg26393629	14			N	1.22	0.24	2.94 × 10^−7^	5.75 × 10^−3^	48.5	Y^4^	N	N	N	N	N	N	
cg19474546	14			Y	−0.71	0.14	3.18 × 10^−7^	5.94 × 10^−3^	30.9	N	N	N	N	N	N	N	
cg04634427	15			N	0.95	0.19	3.39 × 10^−7^	6.07 × 10^−3^	32.5	N	N	N	N	N	N	N	
cg14120919	10	*CRTAC1*	Body	N	0.83	0.16	3.79 × 10^−7^	6.28 × 10^−3^	0.0	N	N	N	N	N	N	N	
cg19529709	22	*TOP1P2*		N	0.51	0.10	3.81 × 10^−7^	6.28 × 10^−3^	47.4	Y	N	N	N	N	N	N	
cg20581874	14			N	0.82	0.16	4.01 × 10^−7^	6.28 × 10^−3^	18.2	Y^4^	N	N	N	N	N	Y	*RNASE6*
cg05371552	7	*VIPR2*	Body	N	1.02	0.20	4.09 × 10^−7^	6.28 × 10^−3^	0.0	Y^3^	N	N	N	N	N	N	
cg07594247	2	*PAX8*		N	1.39	0.27	4.37 × 10^−7^	6.32 × 10^−3^	0.0	Y	N	N	N	N	N	Y	*PAX8/RPL23AP7*
cg27181142	19	*SIX5*	Body	N	0.41	0.08	4.41 × 10^−7^	6.32 × 10^−3^	0.0	N	N	N	Y	N	N	N	
cg17445212	2	*PAX8*		N	1.16	0.23	5.39 × 10^−7^	7.26 × 10^−3^	0.0	N	N	N	N	N	N	Y	*PAX8/RPL23AP7*
cg10678190	16			Y	−0.7	0.14	5.40 × 10^−7^	7.26 × 10^−3^	30.6	Y^3^	N	N	N	N	N	N	
cg21482265	2	*PAX8*		N	1.31	0.26	6.00 × 10^−7^	7.61 × 10^−3^	0.0	N	N	N	N	N	N	Y	*PAX8/RPL23AP7*
**cg12889195^2^**	2	*PAX8*		N	1.45	0.29	6.02 × 10^−7^	7.61 × 10^−3^	0.0	N	N	N	N	N	N	Y	*PAX8/RPL23AP7*
cg07646362	16			N	−0.66	0.13	7.11 × 10^−7^	8.73 × 10^−3^	0.0	N	N	N	N	N	N	N	
cg02679336	10	*FANK1*	Body	N	−0.3	0.06	8.12 × 10^−7^	9.70 × 10^−3^	0.0	Y	N	N	N	N	N	N	
cg27343456	2			N	0.59	0.12	8.52 × 10^−7^	9.75 × 10^−3^	0.0	N	Y	N	N	N	N	N	
cg06882571	16	*GSG1L*		N	−0.49	0.10	8.84 × 10^−7^	9.75 × 10^−3^	0.0	Y	Y	N	N	N	N	N	
cg21540359	7			N	0.67	0.14	8.85 × 10^−7^	9.75 × 10^−3^	0.0	N	N	N	N	N	N	N	
cg02614024	15			N	0.47	0.10	9.18 × 10^−7^	9.87 × 10^−3^	0.0	Y	N	N	N	N	N	N	
cg15612221	18			N	1	0.20	9.55 × 10^−7^	1.00 × 10^−2^	0.0	N	N	N	N	N	N	N	
cg02105458	15			N	0.7	0.14	1.07 × 10^−6^	1.10 × 10^−2^	35.0	N	N	N	N	N	N	N	
cg09317502	7	*HEPACAM2*	TSS1500	Y	−0.71	0.15	1.19 × 10^−6^	1.19 × 10^−2^	11.4	N	N	N	N	N	N	N	
cg22157494	1	*KIAA0562*		N	0.64	0.13	1.22 × 10^−6^	1.19 × 10^−2^	0.0	N	N	N	N	N	N	N	
cg23504719	10	*CRTAC1*	Body	N	0.82	0.17	1.28 × 10^−6^	1.19 × 10^−2^	0.0	N	N	N	N	N	N	N	
cg01809217	1	*GREM2*	Body	N	0.6	0.12	1.28 × 10^−6^	1.19 × 10^−2^	0.0	N	N	N	N	N	N	N	
cg05312960	22	*TOP1P2*		N	0.43	0.09	1.33 × 10^−6^	1.19 × 10^−2^	29.7	N	N	N	N	N	N	N	
cg25843439	19	*OR10H3*	1stExon	Y	−0.54	0.11	1.33 × 10^−6^	1.19 × 10^−2^	25.2	N	N	N	N	N	N	N	
cg13315047	12			N	0.7	0.14	1.42 × 10^−6^	1.22 × 10^−2^	0.0	N	N	N	N	N	N	N	
cg03043822	22	*TOP1P2*		N	0.66	0.14	1.42 × 10^−6^	1.22 × 10^−2^	33.5	Y	N	N	N	N	N	N	
cg23638640	22	*TOP1P2*		N	0.56	0.12	1.59 × 10^−6^	1.34 × 10^−2^	0.0	N	N	N	N	N	N	N	
cg06324373	10	*CRTAC1*	Body	Y	0.96	0.20	1.73 × 10^−6^	1.42 × 10^−2^	0.0	Y^4^	N	N	N	N	N	N	
cg14605520	9	*LHX3*		N	0.79	0.17	1.88 × 10^−6^	1.43 × 10^−2^	25.2	N	N	N	N	N	N	N	
cg08097631	14	*C14orf48*		N	0.51	0.11	1.88 × 10^−6^	1.43 × 10^−2^	0.0	N	N	N	N	N	N	N	
cg01743593	7			N	−0.49	0.10	1.89 × 10^−6^	1.43 × 10^−2^	20.6	N	Y	N	N	N	N	N	
cg01988325	5	*MIR449B*		N	−0.74	0.16	1.89 × 10^−6^	1.43 × 10^−2^	0.0	Y	N	N	N	N	N	N	
cg10340210	4			Y	−0.6	0.13	1.95 × 10^−6^	1.44 × 10^−2^	12.3	Y	Y^6^	N	N	N	N	N	
cg22730830	16	*PRSS21*		N	0.76	0.16	1.99 × 10^−6^	1.44 × 10^−2^	0.0	N	Y	N	N	N	N	Y	*PRSS21*
cg15015426	1	*OR10J5*	TSS1500	Y	−0.75	0.16	2.01 × 10^−6^	1.44 × 10^−2^	0.0	Y	N	N	N	N	N	N	
cg01925498	1			Y	−0.3	0.06	2.16 × 10^−6^	1.49 × 10^−2^	0.0	N	N	N	N	N	N	Y	*LOC645166*
cg11763394	2	*PAX8*		N	1.27	0.27	2.21 × 10^−6^	1.49 × 10^−2^	0.0	N	N	N	N	N	N	Y	*PAX8/RPL23AP7*
cg13174253	8	*C8orf33*	Body	N	−0.47	0.10	2.24 × 10^−6^	1.49 × 10^−2^	26.1	Y	N	N	N	N	N	N	
cg00534274	4	*SLC7A11*		N	0.57	0.12	2.25 × 10^−6^	1.49 × 10^−2^	2.6	N	N	N	N	N	N	N	
cg17465112	1			N	0.61	0.13	2.25 × 10^−6^	1.49 × 10^−2^	0.0	Y	N	N	N	N	N	N	
cg16964673	1	*KIAA0562*		N	0.45	0.10	2.36 × 10^−6^	1.54 × 10^−2^	2.7	N	N	N	N	N	N	N	
cg19480274	20			Y	−0.23	0.05	2.49 × 10^−6^	1.60 × 10^−2^	0.0	N	N	N	N	N	N	N	
cg17234513	15			N	0.74	0.16	3.01 × 10^−6^	1.85 × 10^−2^	19.6	N	N	N	N	N	N	N	
cg24554151	11	*PANX1*	Body	N	−0.7	0.15	3.04 × 10^−6^	1.85 × 10^−2^	5.5	Y^3,4^	N	N	N	N	N	Y	*PANX1*
cg03829137	15			N	0.41	0.09	3.07 × 10^−6^	1.85 × 10^−2^	26.5	N	N	N	N	N	N	N	
cg19479373	10	*CRTAC1*	Body	N	0.67	0.14	3.08 × 10^−6^	1.85 × 10^−2^	0.0	N	N	N	N	N	N	N	
cg04206517	5	*ACSL6*		N	−0.13	0.03	3.18 × 10^−6^	1.87 × 10^−2^	42.9	N	N	N	N	N	N	N	
cg05086444	7	*VIPR2*	Body	N	0.61	0.13	3.55 × 10^−6^	2.06 × 10^−2^	0.0	N	N	N	N	N	N	Y	*PTPRN2*
cg05779786	2	*AGAP1*		N	0.55	0.12	3.83 × 10^−6^	2.12 × 10^−2^	0.0	Y	N	N	N	N	N	N	
cg26536593	1	*ZNF670*	TSS1500	Y	0.71	0.15	3.84 × 10^−6^	2.12 × 10^−2^	28.9	Y^3^	N	N	N	N	N	N	
cg15971980	6			Y	−0.52	0.11	3.90 × 10^−6^	2.12 × 10^−2^	0.0	Y	Y	Y	N	N	N	N	
cg21218093	20	*ZNF335*	5‘UTR	N	−0.37	0.08	4.01 × 10^−6^	2.15 × 10^−2^	0.0	Y	N	N	N	N	N	N	
cg09807524	11			N	0.39	0.09	4.50 × 10^−6^	2.35 × 10^−2^	31.1	N	N	N	N	N	N	N	
cg11258452	12	*SRRM4*	Body	N	0.69	0.15	4.54 × 10^−6^	2.35 × 10^−2^	0.0	N	N	N	N	N	N	N	
cg01613965	2	*LOC654342*	TSS200	Y	−0.34	0.07	4.90 × 10^−6^	2.48 × 10^−2^	2.8	N	N	N	N	N	N	Y	*LOC654342*
cg03689146	7			N	0.75	0.17	4.97 × 10^−6^	2.49 × 10^−2^	0.0	Y	N	N	N	N	N	N	
cg01273232	15			N	0.67	0.15	5.35 × 10^−6^	2.64 × 10^−2^	0.0	Y	N	N	N	N	N	N	
cg06178315	1	*KIAA0562*		N	0.52	0.11	5.48 × 10^−6^	2.68 × 10^−2^	0.0	N	N	N	N	N	N	N	
cg27353899	3	*MUC4*		N	1.37	0.30	5.68 × 10^−6^	2.75 × 10^−2^	4.1	Y	N	N	N	N	N	Y	*MUC20*
cg22809920	20	*RBPJL*		N	0.42	0.09	5.84 × 10^−6^	2.79 × 10^−2^	20.6	Y	N	N	N	N	N	N	
cg17270081	9	*LHX3*		N	0.62	0.14	6.11 × 10^−6^	2.88 × 10^−2^	0.0	N	N	N	N	N	N	N	
cg15730180	7			N	−0.47	0.10	6.17 × 10^−6^	2.88 × 10^−2^	38.8	N	Y^6^	N	N	N	N	N	
cg04579415	6	*TCTE1*	Body	Y	−0.32	0.07	6.75 × 10^−6^	3.09 × 10^−2^	0.0	N	Y^6^	N	N	N	N	N	
cg27403609	2			N	0.38	0.08	7.03 × 10^−6^	3.18 × 10^−2^	23.2	N	N	N	N	N	N	N	
cg08761659	16	*OR1F1*	TSS200	Y	−0.41	0.09	7.27 × 10^−6^	3.26 × 10^−2^	0.0	N	N	N	N	N	N	N	
cg06671706	8	*CLDN23*	1stExon	N	0.71	0.16	7.43 × 10^−6^	3.29 × 10^−2^	0.0	Y	N	N	N	N	N	Y	*RPL23AP7*
cg05010260	7	*C7orf52*	5‘UTR	N	0.49	0.11	7.66 × 10^−6^	3.36 × 10^−2^	0.0	Y^3,4^	N	N	N	N	N	N	
cg16991589	13	*PABPC3*	TSS1500	N	1.13	0.25	7.78 × 10^−6^	3.38 × 10^−2^	0.0	NA	N	N	N	N	N	N	
cg07091678	10	*CRTAC1*	Body	N	0.68	0.15	7.98 × 10^−6^	3.43 × 10^−2^	0.0	Y	N	N	N	N	N	N	
cg17780447	19			N	0.65	0.14	8.25 × 10^−6^	3.51 × 10^−2^	0.0	Y^3^	N	N	N	N	N	N	
cg00074818	8	*CLDN23*	1stExon	N	0.47	0.11	8.93 × 10^−6^	3.76 × 10^−2^	0.0	Y	N	N	N	N	N	Y	*CLDN23*
cg08930881	10	*CRTAC1*	Body	N	0.61	0.14	9.32 × 10^−6^	3.89 × 10^−2^	0.0	N	N	N	N	N	N	N	
cg13605615	1	*DIRAS3*	TSS1500	N	0.32	0.07	9.40 × 10^−6^	3.89 × 10^−2^	0.0	N	N	N	N	N	N	N	
cg27539527	7			N	0.56	0.13	9.51 × 10^−6^	3.90 × 10^−2^	39.6	Y^3^	N	N	N	N	N	N	
cg26434090	11	*DSCAML1*	Body	N	0.47	0.11	9.68 × 10^−6^	3.93 × 10^−2^	0.0	N	N	N	N	N	N	N	
cg18914258	4			Y	−0.33	0.07	1.02 × 10^−5^	4.05 × 10^−2^	0.0	N	N	N	N	N	N	N	
cg01232511	16	*PRSS21*		N	0.83	0.19	1.02 × 10^−5^	4.05 × 10^−2^	0.0	N	Y	N	N	N	N	Y	*PRSS21*
cg15574301	5	*PDLIM4*		N	0.67	0.15	1.03 × 10^−5^	4.05 × 10^−2^	0.0	N	N	N	N	N	N	N	
cg01330954	16			N	−0.48	0.11	1.04 × 10^−5^	4.08 × 10^−2^	0.0	N	N	N	N	N	N	N	
cg21610815	2	*PAX8*		N	0.41	0.09	1.08 × 10^−5^	4.16 × 10^−2^	0.0	N	N	N	N	N	N	N	
cg07280731	16	*PRSS21*		N	0.73	0.17	1.08 × 10^−5^	4.16 × 10^−2^	0.0	Y	N	N	N	N	N	Y	*PRSS21*
cg00466136	11	*NTM*	Body	N	0.66	0.15	1.14 × 10^−5^	4.29 × 10^−2^	0.0	N	N	N	N	N	N	N	
cg08935125	16	*CLDN6*	5‘UTR	N	0.39	0.09	1.14 × 10^−5^	4.29 × 10^−2^	40.2	N	N	N	N	N	N	N	
cg05146852	11	*ANO3*	Body	N	−0.34	0.08	1.17 × 10^−5^	4.29 × 10^−2^	0.0	N	N	Y	N	N	N	N	
cg04453501	7			Y	0.73	0.17	1.18 × 10^−5^	4.29 × 10^−2^	0.0	Y	N	N	N	N	N	N	
cg09520393	4	*DRD5*	TSS200	Y	−0.28	0.06	1.18 × 10^−5^	4.29 × 10^−2^	7.0	N	Y	N	N	N	N	N	

Abbreviations: B12, vitamin B12; *e*QTM: cis-expression quantitative trait methylation; CpG, cytosine-phosphate-guanine site; FDR, false discovery rate; UCSC RefGene, University of California Santa Cruz Reference Gene, SDS, standard deviation score.

1. Coefficients and standard errors represent the percentage change in DNA methylation per SDS increase in vitamin B12. One SDS vitamin B12 corresponded to a weighed mean of 88.8 pmol/L. The prioritized CpGs included those CpGs with FDR-corrected *P*-value<0.05 and little evidence for heterogeneity (I^2^ < 50%).

2. In bold, the CpG with the largest effect size.

3. Bonferroni-significant in childhood look-up (*P*-value<0.05/108 tests; i.e., *P*-value<4.63 × 10–4).

4. Bonferroni-significant in childhood look-up in both early and late childhood (*P*-value<0.05/108 tests; i.e., *P*-value<4.63 × 10–4).

5. Look-up in results of meta-analysis of circulating folate concentrations during pregnancy and epigenome-wide cord blood DNA methylation (13).

6. Not epigenome-wide significant (*P*_FDR_>0.05) after adjustment for circulating maternal folate concentrations.

7. Look-up in results of meta-analysis of birth weight and epigenome-wide cord blood DNA methylation (35).

9. Look-up in results of meta-analyses of overall cognitive skills and nonverbal IQ and epigenome-wide cord blood DNA methylation (37).

### Newborn meta-analysis

Newborn vitamin B12 concentrations were associated with differential DNA methylation at 10 CpGs (P_FDR_<0.05, Figures 2–3) in cord blood after adjusting for the same covariates as in the maternal meta-analysis, except gestational age, which we considered a mediator in this case. Among all analysed CpGs, similar numbers of positive and negative associations were observed (Figure 2). We prioritized 7/10 CpGs with I^2^<50% (Table 3). The association with the lowest *P*-value (1.12 × 10^−7^) was observed for cg13863764 (*Dispatched RND transporter family member 3* gene (*DISP3, also known as PTCHD2;* MIM:*611251*)), with a decrease in DNA methylation per SDS (weighted mean 171.2 pmol/L) increase in vitamin B12 of −0.94% (SE: 0.18%). Cg08243619 (*PTCHD2*) had the largest effect size (decrease in DNA methylation per SDS vitamin B12: −1.09%; SE: 0.21%; *P-*value: 2.85 × 10^−7^). **Supplementary Figure S1** shows the QQ plot of the newborn meta-analysis. There was no evidence of genomic inflation (λ = 1.05).

**Table 3.** Prioritized CpGs (*n* = 7) with differential methylation in cord blood in relation to newborn circulating vitamin B12 concentrations sampled in cord blood at birth.^1^.

CpG	Chromosome	Gene (UCSC RefGene)	Gene location (UCSC RefGene)	Flagged	Coefficient	Standard error	*P*-value	FDR-corrected *P*-value	I^2^	Persistence childhood	Overlap with folate hits in previous study ^5^	Overlap with birth weight hits in previous study ^6^	Overlap with gestational age hits in previous study ^7^	Overlap with overall cognitive skills hits in previous study ^8^	Overlap with nonverbal IQ hits in previous study ^8^	eQTM
cg13863764	1	*PTCHD2*	Body	N	−0.94	0.18	1.12 × 10^−7^	2.96 × 10^−2^	0	Y^3,4^	N	N	N	N	N	N
cg00658405	19	*ADAMTSL5*	Body	N	0.81	0.16	2.79 × 10^−7^	2.96 × 10^−2^	0	N	N	N	N	N	N	N
**cg08243619^2^**	1	*PTCHD2*	Body	N	−1.09	0.21	2.85 × 10^−7^	2.96 × 10^−2^	10.2	Y	N	N	N	Y	Y	N
cg24371425	1	*PTCHD2*	Body	N	−1.03	0.21	9.36 × 10^−7^	4.64 × 10^−2^	0	Y^3^	N	Y	N	Y	N	N
cg02615136	3	*ARIH2*	Body	N	−0.26	0.05	9.93 × 10^−7^	4.64 × 10^−2^	0	N	N	N	N	N	N	N
cg04096723	1	*RERE*		N	−0.28	0.06	1.09 × 10^−6^	4.64 × 10^−2^	0	N	N	N	N	N	N	N
cg09573658	1	*PTCHD2*	Body	N	−0.75	0.15	1.12 × 10^−6^	4.64 × 10^−2^	43.4	Y^3^	N	N	N	N	N	N

Abbreviations: B12, vitamin B12; eQTM: cis-expression quantitative trait methylation; CpG, cytosine-phosphate-guanine site; FDR, false discovery rate; UCSC RefGene, University of California Santa Cruz Reference Gene, SDS, standard deviation score.

1. Coefficients and standard errors represent the percentage change in DNA methylation per SDS increase in vitamin B12. One SDS vitamin B12 corresponded to a weighed mean of 171.2 pmol/L. The prioritized CpGs included those CpGs with FDR-corrected P-value<0.05 and little evidence for heterogeneity (I2 < 50%).

2. In bold, the CpG with the largest effect size.

3. Bonferroni significant in childhood look-up (*P*-value<0.05/7 tests; i.e., *P-*value<0.007).

4. Bonferroni-significant in childhood look-up, in both early and late childhood (*P*-value<0.05/7 tests; i.e., *P*-value<0.007). No persistence of newborn vitamin B12-related methylation was observed into adolescence at this significance level.

5. Look-up in results of meta-analysis of circulating folate concentrations during pregnancy and epigenome-wide cord blood DNA methylation (13).

6. Look-up in results of meta-analysis of birth weight and epigenome-wide cord blood DNA methylation (35).

7. Look-up in results of meta-analysis of gestational age and epigenome-wide cord blood DNA methylation (36).

8. Look-up in results of meta-analyses of overall cognitive skills and nonverbal IQ and epigenome-wide cord blood DNA methylation (37).

### Comparison of maternal and newborn meta-analyses

Epigenome-wide, the correlation between effect estimates from the maternal and newborn meta-analyses was low (*r* = 0.36), but for the prioritized CpGs from both analyses, it was high (*r* > 0.92). In a look-up of the 109 prioritized CpGs from the maternal meta-analysis, 19 (17.6%) of 108 available CpGs were also associated with newborn vitamin B12 concentrations at Bonferroni-significance (*P*-value <0.05/108 tests, i.e., *P*-value <4.63 × 10^−4^; **Supplementary Data 6**), and all had the same direction of effect. In total, 77/108 (71.3%) CpGs were associated with newborn vitamin B12 concentrations with uncorrected *P*-values <0.05 (*P*_enrichment_ = 3.19 × 10^−68^). The seven newborn prioritized CpGs were not associated with maternal vitamin B12 concentrations at Bonferroni significance (*P*-value <0.05/7 tests; i.e., *P*-value <0.007) although one had an uncorrected *P*-value <0.05 and all had the same direction of effect (**Supplementary Data 7**).

### Sensitivity analyses

The leave-one-out analysis for the 109 prioritized CpGs of the maternal meta-analysis showed that no single cohort disproportionately influenced the results. The change in effect estimate when leaving out one cohort was <20% for 83/109 (76%) CpGs. The confidence intervals of all studies overlapped for the 26/109 CpGs with >20% change in effect estimate as shown in **Supplementary Figures S2.1–2.26**. The leave-one-out analysis for the seven prioritized CpGs of the newborn meta-analysis suggested that the much larger GENR was driving the findings (**Supplementary Figures S3.1–3.7**). The direction of the effect was consistent between ALSPAC and GENR for 5/7 prioritized CpGs (**Supplementary Data 8**) [15,16].

Because vitamin B12 concentrations decline during pregnancy, we additionally restricted the maternal meta-analysis to 1,195 (49.4% of total meta-analysis population) mothers from GENR and INMA with vitamin B12 sampled in early pregnancy (<14 weeks gestational age) [8,9,17]. Early-pregnancy associations were largely consistent with anytime associations, with Pearson’s correlation coefficients for the effect estimates *r* = 0.78 (epigenome-wide) and *r* = 0.99 (prioritized CpGs) (**Supplementary Data 9**).

The ATP binding cassette subfamily D member 4 gene (*ABCD4*) gene is involved in the intracellular transport of vitamin B12 [30]. For the prioritized CpGs of the maternal meta-analysis, we tested for interaction between maternal circulating vitamin B12 concentrations and newborn rs3742801 (*ABCD4*) genotype in a meta-analysis among GENR, MoBa1, and MoBa2 [19,20]. None of the interaction terms had uncorrected *P*-values <0.05. This did not justify stratifying the analyses on *ABCD4* genotype.

### Findings of maternal meta-analysis in a multi-ethnic population

Of the 109 prioritized CpGs from the maternal-B12 meta-analysis, cord blood DNA methylation measurements were available in a multi-ethnic population (*n* = 48) from MARBLES for 103 CpGs [18]. Of these, two CpGs were differentially methylated in relation to maternal pregnancy circulating vitamin B12 concentrations (*P*-value <0.05/103 tests, i.e., *P*-value <4.85 × 10^−4^; **Supplementary Data 10**) and 56/103 (54.4%) CpGs had consistent direction of effect.

### Persistence at older ages

We also analysed whether pregnancy and cord blood vitamin B12 concentrations were still associated at Bonferroni-significance with DNA methylation at the CpGs identified in the maternal and newborn meta-analyses in peripheral blood of older children. First, 108/109 prioritized CPSs from maternal meta-analysis were available in GENR and INMA in childhood. In early childhood (ages 4–7 y; *n* = 479), 44 (40.7%) of 108 CpGs were still associated with maternal early-pregnancy circulating vitamin B12 concentrations (*P*-value <0.05/108, i.e., *P*-value <4.63 × 10^−4^). In late childhood (ages 9–10; *n* = 482), 7 (6.5%) CpGs were still associated with maternal early-pregnancy circulating vitamin B12 concentrations (Table 2 and **Supplementary Data 11**). Five CpGs were differentially methylated in children’s blood at both time points. These were cg26393629, cg20581874 (both not close to a gene), cg06324373 (annotated to CRTAC1, Cartilage Acidic Protein 1), cg24554151 (annotated to PANX1, Pannexin 1), and cg05010260 (annotated to C7orf52). The vast majority of the 108 CpGs had a consistent direction of effect as compared to the maternal meta-analysis of newborn DNA methylation (early childhood: 97.2%; late childhood: 91.7%).

Second, in early childhood blood DNA methylation data (*n* = 417, ALSPAC and GENR), 1 (14.3%) of 7 prioritized CpGs of the newborn meta-analysis was still associated with newborn vitamin B12 concentrations (*P*-value <0.05/7; i.e., *P*-value <0.007). In blood DNA methylation data sampled in late childhood (*n* = 321, GENR) and adolescence (age 17; *n* = 83, ALSPAC), 4 (57.1%) and 0 CpGs, respectively, were still associated with newborn vitamin B12 concentrations (**Supplementary Data 12**). The top hit from the meta-analysis of newborn DNA methylation, cg13863764, was still differentially methylated in early and late childhood (Table 3). Most of seven CpGs had consistent directions of effect as compared to the newborn meta-analysis of newborn DNA methylation in early (100%) and late childhood: (85.7%) but not adolescence (42.9%).

### Relationship with folate and homocysteine

The findings of the primary maternal and newborn meta-analysis were largely robust to adjustment for circulating folate concentrations. In the maternal meta-analysis (*n* = 2,397, all cohorts), 89/109 (81.7%) findings remained significant at epigenome-wide level (*P*_FDR_<0.05, **Supplementary Data 9**). Pearson’s correlation coefficient for effect estimates between the primary and folate-adjusted models was high (*r* = 0.99, both epigenome-wide and prioritized CpGs). In the newborn meta-analysis, only GENR had folate concentrations available (*n* = 898). Although all seven findings had *P*_FDR_>0.05, Pearson’s correlation for the effect estimates between the primary and folate-adjusted models was high (*r* = 0.95: epigenome-wide; *r* = 0.99: prioritized CpGs, **Supplementary Data 8**).

We also ran models additionally adjusted for concentrations of homocysteine, an indicator of vitamin B12 status and potential mediator in the identified associations. In the maternal meta-analysis, only the top hit, cg25327343, remained FDR-significant at epigenome-wide level (*n* = 2,020; meta-analysis without INMA; uncorrected *P*-value = 2.31 × 10^−8^; **Supplementary Data 9**). Pearson’s correlation for the effect estimates between the primary and homocysteine-adjusted models was high (epigenome-wide: *r* = 0.85; prioritized CpGs: *r* = 0.99). In newborns, only GENR had homocysteine concentrations available (*n* = 859). Although all seven findings had *P*_FDR_>0.05, Pearson’s correlation coefficient for effect estimates between the primary and homocysteine-adjusted models was high (*r* = 0.90 epigenome-wide; *r* = 0.99: prioritized CpGs, **Supplementary Data 8**).

A hypergeometric test showed that among the 109 prioritized CpGs from the maternal meta-analysis, there were significantly more of the 443 CpGs previously identified for their association with maternal pregnancy circulating folate concentrations than expected by chance, with 15/109 CpGs (13.8%; *P*_enrichment_ = 1.15 × 10^−27^; Table 2) overlapping between the two analyses, which all had the same direction of effect [13]. Of these, 11/15 overlapping CpGs had *P*_FDR_<0.05 after adjustment for vitamin B12 in the previously published folate EWAS meta-analysis [13]. Also, 10/15 had *P*_FDR_<0.05 in our folate-adjusted meta-analysis (**Supplementary Data 9**) [13]. For the newborn meta-analysis, none of the seven identified CpGs were among the 443 hits from the previous folate meta-analysis [13]. The three differentially methylated CpGs reported by a previous meta-analysis of circulating homocysteine concentrations in adults were not among the CpGs with uncorrected *P*-values <0.05 and I^2^<50% in either the maternal or newborn vitamin B12 meta-analysis [24,33]. The prioritized CpGs of the maternal and newborn meta-analyses did not overlap with the three CpGs in cord blood that were previously associated with maternal vitamin B12 concentrations as proxied by maternal fucosyltransferase 2 (*FUT2*-) genotype [12].

### Perinatal and childhood health outcomes

To explore whether the identified CpGs may represent pathways underlying associations of vitamin B12 concentrations during foetal development with child health outcomes, we examined associations of our findings with birth weight and gestational age at birth, and with childhood overall cognitive skills and childhood nonverbal IQ, using previously published EWASs [35–37]. Of the prioritized CpGs in the maternal and newborn meta-analysis, 4/109 (*P*-value <0.05/109 tests; i.e., *P*-value <4.59 × 10^−4^) and 1/7 CpGs (*P*-value <0.05/7 tests; i.e., *P*-value <0.007), respectively, were also differentially methylated in relation to birth weight, with a similar direction of effect (Tables 2–3 and **Supplementary Data 13–14**) [35]. Of the prioritized CpGs in the maternal meta-analysis, 1/109 CpGs (cg27181142) was also differentially methylated in relation to gestational age, with a similar direction of effect (Tables 2–3 and **Supplementary Data 13–14**) [36]. None of the prioritized CpGs in the maternal meta-analysis were differentially methylated in relation to childhood overall cognitive skills and childhood nonverbal IQ (**Supplementary Data 13**) [37]. Of the prioritized CpGs in the newborn meta-analysis, 5/7 were available in the meta-analyses on childhood cognitive skills and childhood nonverbal intelligence [37]. Of these, 1/5 and 2/5 CpGs, respectively, were differentially methylated in relation to these traits, with a similar direction of effect (**Supplementary Data 14**). None of the five CpGs that were differentially methylated in children’s blood in either early or late childhood were associated with childhood health outcomes.

**Follow-up analyses of the identified CpG sites** The 109 and 7 prioritized CpGs from the maternal and newborn meta-analysis, respectively, showed little evidence for functional enrichment of GO terms (smallest *P*-value = 9.8 × 10^−4^) or KEGG terms (smallest *P*-value = 4.9 × 10^−3^) terms (**Supplementary Data 15–19**). For the 109 prioritized CpGs from the maternal meta-analysis, we identified 57 unique CpG-gene expression pairs (*cis*-eQTM) using data from the HELIX project [39]. These *cis*-eQTMs involved 18 unique transcript clusters (equivalent to putative genes) with transcription start sites within ± 500kb of any of 20/109 (18.3%) prioritized CpGs (Tables 1–2; **Supplementary Data 19**). Most associations (41/57, 71.9%) were negative, indicating that higher methylation was associated with lower gene expression. The association with the lowest *P*-value (9.48 × 10^−185^) was observed between methylation at cg21482265 and gene expression of *PAX8*, with a log_2_ fold change in expression per 10% increase in DNA methylation of −0.096 (SE 0.025). DNA methylation at *cg20581874* and *cg24554151* was associated with expression of *RNASE6* (Ribonuclease A Family Member K6) and *PANX1*, respectively. There was no evidence for enrichment in the DNase I hypersensitive sites for the prioritized CpGs of both meta-analyses [40]. Among the 109 prioritized CpGs of the maternal meta-analysis, we observed evidence for enrichment for several chromatin states and histone marks and one transcription factor motif (**Supplementary Figure S4.1–4.2 and 5.1–5.2**).

## Discussion

This comprehensive analysis on the associations of circulating vitamin B12 concentrations during foetal development and epigenome-wide cord blood DNA methylation was a joint effort of six birth cohorts. Maternal pregnancy and newborn circulating vitamin B12 concentrations were associated with differential methylation at 109 and 7 CpGs, respectively, in newborns. We observed persistence for up to 40.7% (44 of 108 available CpGs) of the CpGs associated with maternal vitamin B12 (‘maternal vitamin B12-related CpGs’) and 57.1% (4 of 7 CpGs) of the CpGs associated with newborn vitamin B12 (‘newborn B12-related CpGs’) at school-age. Furthermore, 3.7% (4 of 109 CpGs) and 14.3% (1 of 7 CpGs) of differentially methylated CpGs in the maternal and newborn meta-analysis, respectively, were previously related to birth weight. Also, 0.9% (1 of 109 CpGs) of differentially methylated CpGs in the maternal meta-analysis were previously related to gestational age. Of the differentially methylated CpGs of the newborn meta-analysis, 14.3% (1 of 7 CpGs) and 28.6% (2 of 7 CpGs), respectively, were previously related to childhood cognitive skills and childhood nonverbal intelligence. Associations with nearby gene expression were observed for 18.3% (20 of 109 CpGs) of maternal vitamin B12-related CpGs.

Vitamin B12 is involved in one-carbon metabolism, which supplies the methyl groups for DNA methylation by guaranteeing the availability of methionine [8]. Differential foetal DNA methylation may underlie the known associations of circulating vitamin B12 concentrations during foetal development with childhood health [2–5,8]. Previously, circulating vitamin B12 concentrations during pregnancy have been associated with global and gene-specific, but not epigenome-wide cord blood DNA methylation [10,11].

We observed associations of maternal circulating vitamin B12 concentrations during pregnancy with differential DNA methylation at 109 CpGs in offspring cord blood. The associations were largely similar in leave-one-out analyses, among mothers with vitamin B12 sampled in early pregnancy only, and when additionally adjusted for circulating folate concentrations. In total, 15/109 CpGs were also differentially methylated in relation to maternal pregnancy folate concentrations in previous work [13]. This meta-analysis used data from MoBa and GENR and thus is not independent from our findings. The overlap emphasizes that not all associations of folate and vitamin B12 with differential cord blood DNA methylation are specific to one of these vitamins. This seems plausible as vitamin B12 and folate closely interact. After adjustment for homocysteine, a functional marker of vitamin B12 status, only the top CpG site still had *P*_FDR_<0.05 (*P*-value = 2.31 × 10^−8^). As this analysis included 16.5% fewer participants, the absolute effect estimates only changed mildly, and the correlations between effect estimates of the findings were high, low power may explain the attenuation of the identified associations. Yet, it may also be biologically plausible that homocysteine, donor of methyl groups and a functional marker of vitamin B12 status, acts as a partial mediator in associations between vitamin B12 and DNA methylation. In one-carbon metabolism, homocysteine is remethylated into methionine via two complementary and interacting, cyclic enzymatic pathways. One of these pathways is folate-dependent and requires vitamin B12 as catalyser [8]. Furthermore, only two of the findings from the maternal meta-analysis were also observed in a look-up among a small, multi-ethnic population of newborns, the MARBLES cohort. The limited replication in this specific population may indicate that not all findings can be extrapolated to participants from non-European ancestry, but it may also simply be due to low power. Thus, further exploration in larger studies and more diverse populations is needed.

The maternal meta-analysis included about twice as many participants as the newborn meta-analysis. Nonetheless, 17.6% of the prioritized maternal vitamin B12-related CpGs were also differentially methylated in a look-up in the newborn meta-analysis and 71.3% had an uncorrected *P*-value <0.05. Additionally, all prioritized CpGs had consistent directions of association across both meta-analyses, and effect estimates were highly correlated. Furthermore, we observed persistence of a large number of maternal vitamin B12-related differentially methylated CpGs across childhood. Thus, we may have been underpowered in the newborn meta-analysis to find associations at the same loci as those identified in the maternal meta-analysis. Based on our findings, we consider it likely that this explains the majority of the differences between maternal and newborn meta-analyses. Alternatively or additionally, maternal and offspring circulating vitamin B12 concentrations may be associated with cord blood DNA methylation at different CpGs. Maternal and newborn vitamin B12 concentrations seem to correlate moderately [41]. The placenta produces transcobalamin II, a protein that can bind vitamin B12. This complex is called active B12 and is the metabolically active form of vitamin B12. Vitamin B12 is actively transported from mother to foetus by specific placental transport-carriers. These bind active B12 from maternal blood and transport it to foetal circulation. The placenta can further regulate foetal vitamin B12 uptake by changing the rate of cobalamin II synthesis [8,41,42]. Furthermore, it has to be noted that as maternal vitamin B12 was sampled in the first half of pregnancy and offspring samples were taken at birth, associations may be time-specific. This may be plausible from a biological perspective. Vitamin B12 concentrations vary in different stages of pregnancy [41]. Vitamin B12 has been associated with neurodevelopment. This complex, dynamic process involves precisely orchestrated molecular and cellular events [8,12,43]. For the hypothesized inverse association with neural tube defects, vitamin B12 status during early pregnancy seems more relevant than during late pregnancy [41]. Also, inconsistent associations with child cardiometabolic outcomes have been previously reported for maternal versus newborn circulating vitamin B12 concentrations [2,44]. Finally, the different findings for maternal and newborn meta-analyses may be explained by differences between the cohorts included in both meta-analyses, such as vitamin B12 intake from diet or multivitamins, which might be used less frequently in late pregnancy, as compared to early pregnancy. Yet, a previous meta-analysis of EWAS on vitamin B12 dietary intake in 5,841 adults reported no association with methylation in leukocytes [45].

Associations of multiple maternal and newborn vitamin B12-related CpGs in newborns persisted in childhood. The CpGs that were differentially methylated at both time points in childhood were annotated to *CRTAC1*, *PANX1,* and *C7orf52*. Two of them, *cg20581874* and *cg24554151,* were associated with the expression of *RNASE6* (Ribonuclease A Family Member K6) and *PANX1*, respectively. *CRTAC1* encodes a glycosylated extracellular matrix protein that is found in the interterritorial matrix of cartilage. It may be involved in cell–cell or cell–matrix interactions. *PANX1* belongs to the innexin family and is a structural component of gap junctions. This protein is abundantly expressed in the central nervous system. *C7orf52* is predicted to enable acyl-transferase activity. *RNASE6* plays a role in the urinary tract [46]. We had no data to examine whether maternal vitamin B12-related differential methylation persisted into adolescence. Such persistence was not observed for the prioritized CpGs from the newborn meta-analysis, but we only had data on 83 adolescents. Thus, this could be power related. Alternatively, differential DNA methylation at birth may return to normal levels between late childhood and adolescence. Persistence of differential methylation is not a prerequisite for effects on long-term health. Vitamin B12-related differential DNA methylation during organogenesis may induce early functional or structural alterations that cannot be reversed, regardless of the persistence of differential DNA methylation itself. However, studies on causality in these associations are needed. Whether postnatal exposures, such as infant vitamin B12 intake, could modify associations of vitamin B12-related differential methylation with child health also needs further study [47].

We observed that 3.7% and 14.3% of prioritized CpGs of the maternal and newborn meta-analysis, respectively, were also differentially methylated in relation to birth weight in a previous meta-analysis of EWASs [35]. One prioritized CpG of the maternal meta-analysis was also differentially methylated in relation to gestational age at birth in another meta-analysis of EWASs [36]. A previous meta-analysis of observational studies reported associations of maternal pregnancy vitamin B12 deficiency with higher risk of low birth weight and prematurity [4]. Of the differentially methylated CpGs of the newborn meta-analysis, 14.3% and 28.6%, respectively, were also differentially methylated in relation to childhood cognitive skills and childhood nonverbal intelligence in a previous meta-analysis of EWASs [37]. Previously, a Mendelian randomization study suggested a causal role for DNA methylation in the association of maternal circulating vitamin B12 concentrations during pregnancy with child IQ [12]. Combined, these findings suggest that vitamin B12-related differential DNA methylation may underlie associations of vitamin B12 concentrations during foetal life with foetal and childhood growth and development, but further studies examining these pathways in more detail need to be done.

We identified 57 *cis*-eQTMs among CpGs with *P*_FDR_<0.05 in the maternal meta-analysis. Of these, 18 eQTMs, including 6 unique CpGs and 3 unique transcript clusters, mapped to *PAX8* and showed an inverse association. Seven prioritized CpGs of the maternal meta-analysis were also annotated to *PAX8*. Associations annotated to *PAX8* in general had relatively large effect sizes and showed increased methylation in relation to higher vitamin B12 concentrations. Thus, higher vitamin B12 concentrations seem to be associated with lower expression of *PAX8*. *PAX8* belongs to the paired box family of transcription factors and is involved in thyroid follicular cell development and expression of thyroid-specific genes [48]. Thyroid hormones are essential for normal foetal growth and development [49]. Our findings annotated to *PAX8* were not associated with birth weight [35]. The top hit of the maternal meta-analysis, cg25327343, located in the gene body of *MAL2*, was not associated with nearby gene expression. *MAL2* encodes a transmembrane protein belonging to the *MAL* proteolipid family. It is part of glycolipoprotein lipid microdomains of cell membranes and required for intracellular polarized transport [50]. This particular CpG has been associated with foetal brain development and excessive alcohol consumption during pregnancy [51–53]. In the newborn meta-analysis, 4/7 prioritized CpGs mapped to the body of *PTCHD2* (also known as DISP3). All were negatively associated with vitamin B12. *PTCHD2* encodes a 13-transmembrane domain-containing protein and is regulated by thyroid hormone [54]. It is highly expressed in neural tissue and involved in neuronal proliferation and differentiation, and cholesterol metabolism [48,55]. Taken together, the examination of potential functional relevance of the identified CpG sites in this work revealed that 18% of the identified CpGs may be associated with gene expression. In addition, there was evidence for enrichment of several chromatin states and histone marks and one transcription factor motif. These findings indicate that the identified CpGs may indeed have functional consequences. However, further experimental work would be needed to examine this in more detail and establish the exact role of DNA methylation in these CpGs in biological processes associated with vitamin B12 metabolism.

The comprehensive design of this study is a major strength. We used data from a large number of participants from prospective birth cohorts and information on circulating vitamin B12 concentrations at two stages during foetal development. We also had repeated DNA methylation measurements available for a substantial number of children. Importantly, the participants included in this analysis were from high-income countries and from relatively high socioeconomic backgrounds. This limits the generalizability of the observed associations to populations from less wealthy countries or people from different socioeconomic backgrounds. The observed associations had small effect estimates. Yet, small changes in DNA methylation levels may still have biological effects [56]. We assessed DNA methylation in cord blood, which is relatively easy to collect. However, DNA methylation is tissue-specific. Other tissues may be more relevant for health outcomes, such as brain, which is not available in population-based studies for obvious reasons [8]. Also, as in any observational study, residual confounding may be present, despite adjustment for relevant confounders. In addition, the Illumina 450k array only covers around 2% of all CpGs across the genome. Vitamin B12 concentrations during development may be associated with DNA methylation in non-measured CpGs.

## Conclusions

In summary, we showed that maternal and newborn vitamin B12 concentrations are associated with DNA methylation at multiple CpGs in offspring blood, many of which are persistent into childhood and some of which were previously associated with relevant phenotypes, including birth weight, gestational age, and cognitive skills. DNA methylation in a number of the identified CpGs was associated with gene expression of nearby genes in an external dataset. Whether DNA methylation at these CpG sites mediates associations of vitamin B12 concentrations with child health outcomes should be further examined in future studies.

## Supplementary Material

Supplemental Material

## Data Availability

Analysis plan and R code for cohort-specific analyses and meta-analyses are available via https://github.com/GiuliettaMonasso/PACE-B12-meta-analysis-of-EWAS. The dataset(s) supporting the conclusions of this article is available in the [Zenodo repository]. All further relevant data supporting the key findings of this study are available within the article and its Supplementary Information files or from the corresponding author upon reasonable request and subject to the study-specific data access procedures. Requests for access to the individual-level data for ALSPAC can be directed to GCS: gemma.sharp@bristol.ac.uk. Requests for access to the individual-level data for GENR can be directed to JFF: j.felix@erasmusmc.nl. Requests for access to the individual-level data for INMA can be directed to MB: mariona.bustamante@isglobal.org. Requests for access to the individual-level data for MARBLES can be directed to RJS: rjschmidt@ucdavis.edu. Requests for access to the individual-level data for MoBa1 and MoBa2 can be directed to SEH: SiriEldevik.Haberg@fhi.no.

## List of abbreviations

Adj: adjusted
ALSPAC: the Avon Longitudinal Study of Parents and Children
cis-eQTM: cis-expression quantitative trait methylation
EWAS: epigenome-wide association study
FDR: False Discovery Rate
GENR: the Generation R Study
GO: Gene Ontology
HELIX: Human Early-Life Exposome
INMA: the Sabadell subcohort of the INfancia y Medio Ambiente Project
KEGG: Kyoto Encyclopedia of Genes and Genomes
MARBLES: the Markers of Autism Risk Learning Early Signs
MoBa1: Norwegian Mother Father and Child Cohort Study
SD: standard deviation
SDS: standard-deviation-score
SNP: single nucleotide polymorphism
