# Supplementary material for: A meta-analysis of epigenome-wide association studies on pregnancy vitamin B12 concentrations and offspring DNA methylation
Source: Epigenetics. 2023 Apr 24;18(1):2202835. doi: 10.1080/15592294.2023.2202835 (PMC10128528; doi:10.1080/15592294.2023.2202835)
Supplement: Supplemental Material [file KEPI_A_2202835_SM7154.zip › Supplementary files/Acknowledgements_PACE_B12.docx]

**Circulating vitamin B12 concentrations during pregnancy and cord blood DNA methylation: a meta-analysis of epigenome-wide association studies**

**Acknowledgements**

**Avon Longitudinal Study of Parents and Children (ALSPAC)**

We are extremely grateful to all the families who took part in this study, the midwives for their help in recruiting them, and the whole ALSPAC team, which includes interviewers, computer and laboratory technicians, clerical workers, research scientists, volunteers, managers, receptionists, and nurses. Please note that the ALSPAC study website (http://www.bristol.ac.uk/alspac/researchers/our-data/) contains details of all the data that is available through a fully searchable data dictionary and variable search tool. We would like to acknowledge Tom Gaunt, Oliver Lyttleton, Sue Ring, Nabila Kazmi, and Geoff Woodward for their earlier contribution to the generation of ARIES data (ALSPAC methylation data).

**Generation R (GENR)**

The Generation R Study is conducted by Erasmus MC, University Medical Center Rotterdam in close collaboration with the School of Law and Faculty of Social Sciences of the Erasmus University Rotterdam, the Municipal Health Service Rotterdam area, Rotterdam, the Rotterdam Homecare Foundation, Rotterdam and the Stichting Trombosedienst & Artsenlaboratorium Rijnmond (STAR-MDC), Rotterdam. We gratefully acknowledge the contribution of children and parents, general practitioners, hospitals, midwives and pharmacies in Rotterdam. The study protocol was approved by the Medical Ethical Committee of Erasmus MC. Written informed consent was obtained for all participants. The generation and management of the Illumina 450K methylation array data (EWAS data) for the Generation R Study was executed by the Human Genotyping Facility of the Genetic Laboratory of the Department of Internal Medicine, Erasmus MC, and the Netherlands. We thank Mr. Michael Verbiest, Ms. Mila Jhamai, Ms. Sarah Higgins, Mr. Marijn Verkerk and Dr. Lisette Stolk for their help in creating the EWAS database. We thank Dr. A.Teumer for his work on the quality control and normalization scripts.

**INfancia y Medio Ambiente** **(INMA) - Sabadell**

The authors would particularly like to thank all the participants for their generous collaboration. A full roster of the INMA Project Investigators can be found at <http://www.proyectoinma.org/presentacion-inma/listado-investigadores/en_listado-investigadores.html>.

**Markers of Autism Risk Learning Early Signs (MARBLES)**

We are grateful to the participants of the MARBLES Study, without whom this work would not have been possible.

**Norwegian Mother, Father and Child Cohort Study (MoBa): MoBa1 and MoBa2**

We are grateful to all the participating families in Norway who took part in this on-going cohort study. We thank Jakob Mjånes and Elin Alsaker of the National Institute of Public Health (Bergen, Norway), Dr. Frank Day of NIEHS and Dr. Jianping Jin of Westat (Durham, NC) for expertise in data management and computational assistance.
