# Supplementary material for: A meta-analysis of epigenome-wide association studies on pregnancy vitamin B12 concentrations and offspring DNA methylation
Source: Epigenetics. 2023 Apr 24;18(1):2202835. doi: 10.1080/15592294.2023.2202835 (PMC10128528; doi:10.1080/15592294.2023.2202835)
Supplement: Supplemental Material [file KEPI_A_2202835_SM7154.zip › Supplementary files/Funding_PACE_B12l.docx]

**Circulating vitamin B12 concentrations during pregnancy and cord blood DNA methylation: a meta-analysis of epigenome-wide association studies**

**Funding**

**Avon Longitudinal Study of Parents and Children (ALSPAC)**

GM and GCS are members of the MRC Integrative Epidemiology Unit, which receives funds from the University of Bristol and United Kingdom Medical Research Council [MC_UU_00011/1, MC_UU_00011/5 and MC_UU_00011/6]. GCS’s contribution to this work is supported by the Medical Research Council [New Investigator Research Grant, MR/S009310/1]. GM, LJ and GCS’s contributions are supported by the European Joint Programming Initiative “A Healthy Diet for a Healthy Life” (JPI HDHL, NutriPROGRAM project, UK MRC MR/S036520/1]. The UK Medical Research Council and the Wellcome Trust (Grant ref: 217065/Z/19/Z) and the University of Bristol provide core support for ALSPAC. A comprehensive list of grants funding is available on the ALSPAC website (http://www.bristol.ac.uk/alspac/external/documents/grant-acknowledgements.pdf). The Accessible Resource for Integrated Epigenomics Studies (ARIES) which generated large scale methylation data was funded by the UK Biotechnology and Biological Sciences Research Council (BB/I025751/1 and BB/I025263/1). Additional epigenetic profiling on the ALSPAC cohort was supported by the UK Medical Research Council Integrative Epidemiology Unit and the University of Bristol (MC_UU_12013_1, MC_UU_12013_2, MC_UU_12013_5 and MC_UU_12013_8), the United States National Institute of Health (5RO1AI121226-02) and National Institute of Child and Human Development grant (R01HD068437). The funders had no role in study design, data collection and analysis, decision to publish, or preparation of the manuscript. This publication is the work of the authors and Gemma Sharp will serve as guarantors for the contents of this paper. The views expressed in this paper are those of the authors and not necessarily any funders. The funders had no influence on the content of the paper.

**Generation R (GENR)**

The general design of the Generation R Study is made possible by ﬁnancial support from the Erasmus MC, University Medical Centre Rotterdam, Erasmus University Rotterdam, the Netherlands Organization for Health Research and Development (ZonMw), the Netherlands Organization for Scientific Research (NWO), the Ministry of Health, Welfare and Sport and the Ministry of Youth and Families. The EWAS data was funded by a grant to VWVJ from the Netherlands Genomics Initiative (NGI)/Netherlands Organization for Scientific Research (NWO) Netherlands Consortium for Healthy Aging (NCHA; project number 050-060-810), by funds from the Genetic Laboratory of the Department of Internal Medicine, Erasmus MC, and by a grant from the National Institute of Child and Human Development (R01HD068437). VWVJ received funding from the European Research Council (ERC-2014-CoG-648916). The project was supported by funding from the European Union's Horizon 2020 research and innovation program under grant agreements No 733206 (LifeCycle), 874739 (LongITools) and 824989 (EUCAN-Connect), and from the European Joint Programming Initiative “A Healthy Diet for a Healthy Life” (JPI HDHL, NutriPROGRAM project, ZonMw the Netherlands no.529051022 and PREcisE project ZonMw the Netherlands no.529051023).

**INfancia y Medio Ambiente** **(INMA) - Sabadell**

This study was funded by grants from Instituto de Salud Carlos III (Red INMA G03/176; CB06/02/0041; PI041436), PI081151 incl. FEDER funds), Generalitat de Catalunya-CIRIT 1999SGR 00241, Fundació La marató de TV3 (090430), the European Community’s Seventh Framework Programme under grant agreements no 308333 (HELIX project) and 261357 (MEDALL project), the European Community’s Seventh Framework Programme - European Research Council (ERC) grant agreement no 268479 (BREATHE Project), the European Union’s Horizon 2020 research and innovation programme under grant agreement no 733206 (LIFECYCLE), and the European Joint Programming Initiative “A Healthy Diet for a Healthy Life” (JPI HDHL and Instituto de Salud Carlos III) under the grant agreement no AC18/00006 (NutriPROGRAM project).

We acknowledge support from the Spanish Ministry of Science and Innovation through the “Centro de Excelencia Severo Ochoa 2019-2023” Program [CEX2018-000806-S], and support from the Generalitat de Catalunya through the CERCA Program.

**Markers of Autism Risk Learning Early Signs (MARBLES)**

The MARBLES study and this work has been supported by a grant from the Allen Foundation, pilot funding from the MIND Institute, EPA STAR grant #RD-83329201, and NIH grants: R24ES028533, R01ES028089, R01ES020392, R01ES025574, P01ES011269, and K12HD051958. These supporting organizations had no role in the design and conduct of the work; collection, management, analysis, and interpretation of the data; preparation, review, or approval of the manuscript; and decision to submit the manuscript for publication. The findings and conclusions in this report are those of the authors and do not necessarily represent the official position of the National Institutes of Health or EPA.

**Norwegian Mother, Father and Child Cohort Study (MoBa): MoBa1 and MoBa2**

The Norwegian Mother and Child Cohort Study are supported by the Norwegian Ministry of Health and Care Services and the Ministry of Education and Research, NIH/NIEHS (contract no N01-ES-75558), NIH/NINDS (grant no.1 UO1 NS 047537-01 and grant no.2 UO1 NS 047537-06A1). For this work, MoBa 1 and 2 were supported by the Intramural Research Program of the NIH, National Institute of Environmental Health Sciences (Z01-ES-49019) and the Norwegian Research Council/BIOBANK (grant no 221097). This work was partly supported by the Research Council of Norway through its Centres of Excellence funding scheme, project number 262700.
