# Supplementary material for: A meta-analysis of epigenome-wide association studies on pregnancy vitamin B12 concentrations and offspring DNA methylation
Source: Epigenetics. 2023 Apr 24;18(1):2202835. doi: 10.1080/15592294.2023.2202835 (PMC10128528; doi:10.1080/15592294.2023.2202835)
Supplement: Supplemental Material [file KEPI_A_2202835_SM7154.zip › Supplementary files/Supplementary information.docx]

**Additional files**

**Supplementary Figures**

 Supplementary Figure 1. QQ plots of the maternal and newborn meta-analysis.

 Supplementary Figures 2.1-2.26. Forest plots of CpGs with >20% change in effect estimate in leave-one-out analyses: maternal-B12 meta-analysis.

 Supplementary Figures 3.1-3.7. Forest plots of 7 prioritized CpGs in leave-one-out analyses: newborn-B12 meta-analysis.

 Supplementary Figures 4.1-4.2. Enrichment of 109 maternal prioritized CpGs for chromatin states and histone marks.

 Supplementary Figures 5.1-5.2. Enrichment of 109 maternal prioritized CpGs (upper panel) and 7 newborn prioritized CpGs (lower panel) for transcription factor motifs.

**Supplementary Note (.pdf)**

 Accession code full results (all models)

 Supplementary Methods. Cohort-specific and in alphabetical order

 Supplementary Acknowledgements. Cohort-specific and in alphabetical order

 Supplementary Funding. Cohort-specific and in alphabetical order

 Supplementary References.

**Supplementary Data (.xlsx)**

 Supplementary Data 1. Cohort-specific methods.

 Supplementary Data 2. Cohort-specific characteristics of participants in analyses of maternal circulating vitamin B12 concentrations during pregnancy.

 Supplementary Data 3. Cohort-specific characteristics of participants in analyses of newborn circulating vitamin B12 concentrations.

 Supplementary Data 4. Pearson's correlation of one-carbon metabolism markers per study.

 Supplementary Data 5. Genomic inflation factors (lambdas) per cohort and for all meta-analyses.

 Supplementary Data 6. Results for 109 prioritized CpGs of maternal meta-analysis in newborn meta-analysis.

 Supplementary Data 7. Results for 7 prioritized CpGs of newborn meta-analysis in maternal meta-analysis.

 Supplementary Data 8. Results for 7 prioritized CpGs of newborn meta-analysis (sensitivity and secondary analyses).

 Supplementary Data 9. Results for 109 prioritized CpGs of maternal meta-analysis (sensitivity and secondary analyses).

 Supplementary Data 10. Loop-up of 109 prioritized CpGs of maternal meta-analysis in newborns from multi-ethnic population.

 Supplementary Data 11. Look-up of 109 prioritized CpGs of maternal meta-analysis in early and late childhood.

 Supplementary Data 12. Look-up of 7 prioritized CpGs of newborn meta-analysis in early and late childhood and adolescence.

 Supplementary Data 13. Look-up of 109 prioritized CpGs of maternal meta-analysis in in meta-analyses of EWAS on birth weight, gestational age, child overall cognitive skills and child nonverbal IQ.

 Supplementary Data 14. Look-up of 7 prioritized CpGs of newborn meta-analysis in meta-analyses of EWAS on birth weight, gestational age, child overall cognitive skills and child nonverbal IQ

 Supplementary Data 15. Enrichment analysis for 109 prioritized CpGs of maternal meta-analysis (GO data source).

 Supplementary Data 16. Enrichment analysis for 109 prioritized CpGs of maternal meta-analysis (KEGG data source).

 Supplementary Data 17. Enrichment analysis for 7 prioritized CpGs of newborn meta-analysis (GO data source).

 Supplementary Data 18. Enrichment analysis for 7 prioritized CpGs of newborn meta-analysis (KEGG data source).

 Supplementary Data 19. Associations of 109 prioritized CpGs of maternal meta-analysis with nearby gene expression.
