# Supplementary material for: A meta-analysis of epigenome-wide association studies on pregnancy vitamin B12 concentrations and offspring DNA methylation
Source: Epigenetics. 2023 Apr 24;18(1):2202835. doi: 10.1080/15592294.2023.2202835 (PMC10128528; doi:10.1080/15592294.2023.2202835)
Supplement: Supplemental Material [file KEPI_A_2202835_SM7154.zip › Supplementary files/Supplementary_Information_PACE_B12_final.docx]

**Circulating vitamin B12 concentrations during pregnancy and cord blood DNA methylation: a meta-analysis of epigenome-wide association studies**

**Supplementary information**

Giulietta S. Monasso, Thanh T. Hoang, Giulia Mancano, Sílvia Fernández-Barrés, John Dou, Vincent W.V. Jaddoe, Christian M. Page, Laura Johnson, Mariona Bustamante, Kelly M. Bakulski, Siri E. Håberg, Per M. Ueland, Thomas Battram, Simon K. Merid, Erik Melén, Doretta Caramaschi, Leanne K. Küpers, Jordi Sunyer, Wenche Nystad, Sandra G. Heil, Rebecca J. Schmidt , Martine Vrijheid, Gemma C. Sharp^4,5^, Stephanie J. London^3^, Janine F. Felix

**
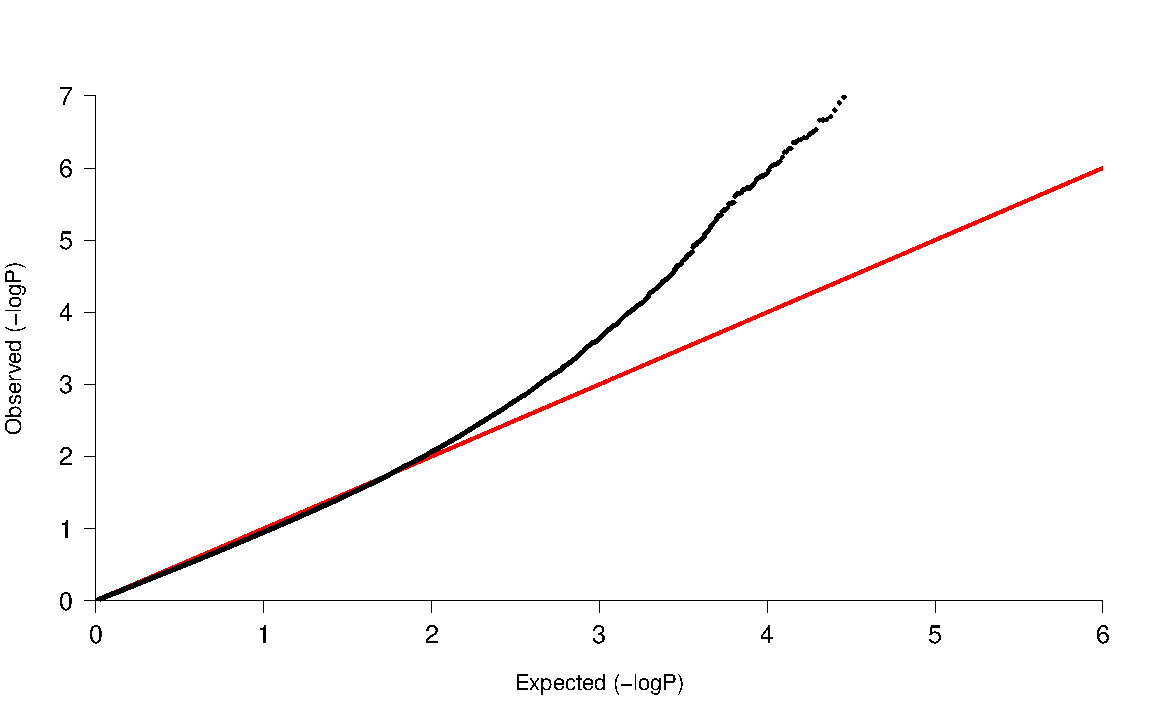
**

λ = 0.98

λ = 1.05

**Supplementary Figure 1. QQ plots of the maternal-B12 and newborn-B12 meta-analysis.**

Upper panel shows QQ plot of maternal-B12 meta-analysis. Lower panel shows QQ plot of newborn-B12 meta-analysis. Lambdas of all cohort-specific analyses are provided in **Supplementary Table 4**.

**
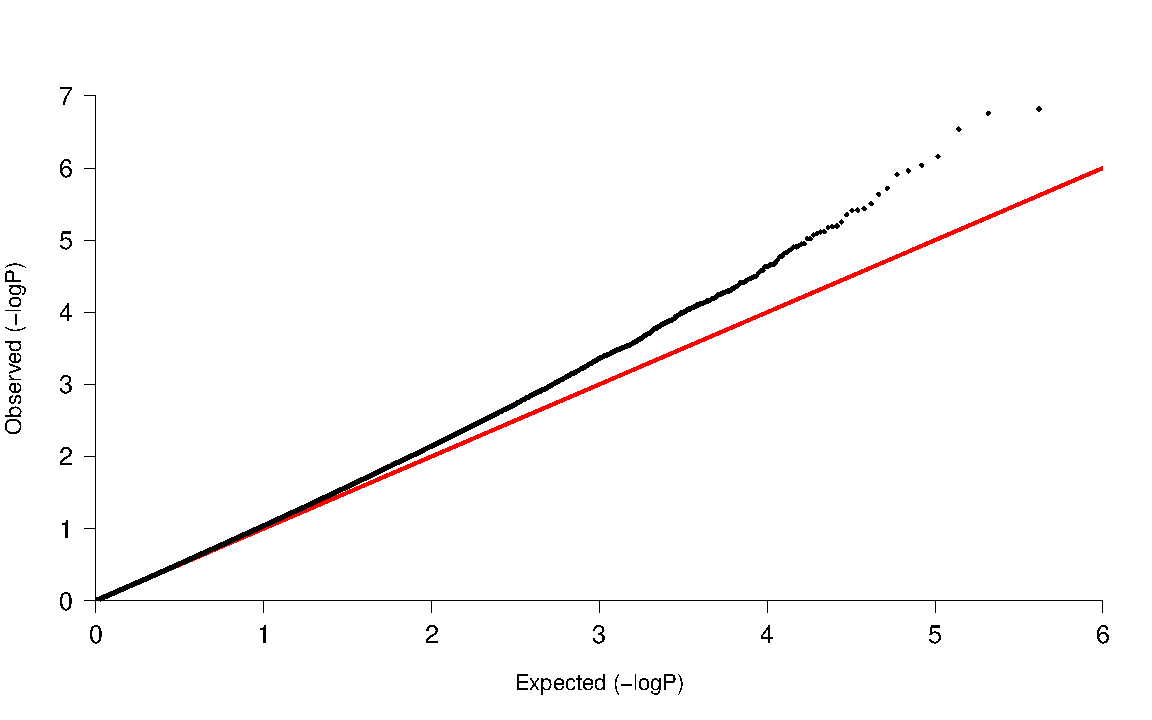
**

**
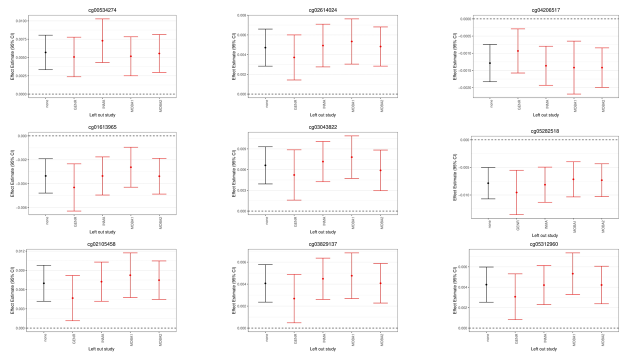
**

**
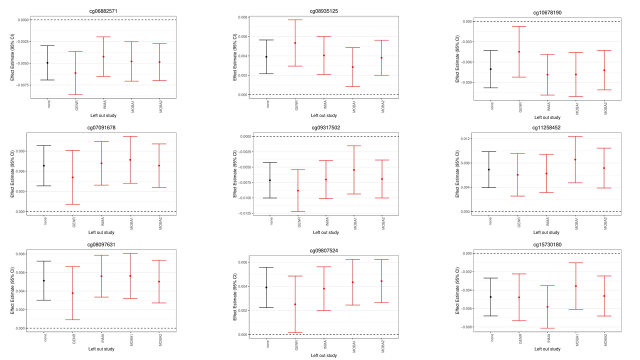
**

**
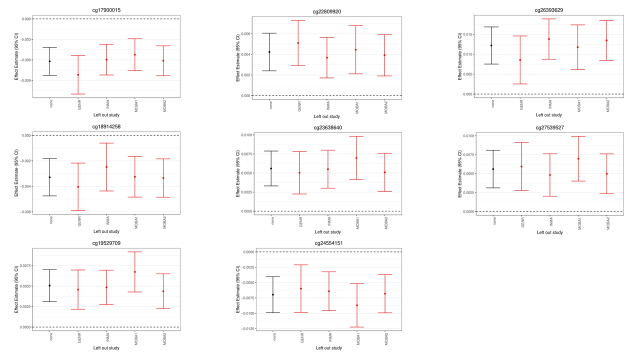
**

**Supplementary Figures 2.1-2.26. Forest plots of CpGs with >20% change in effect estimate in leave-one-out analyses: maternal-B12 meta-analysis**

**
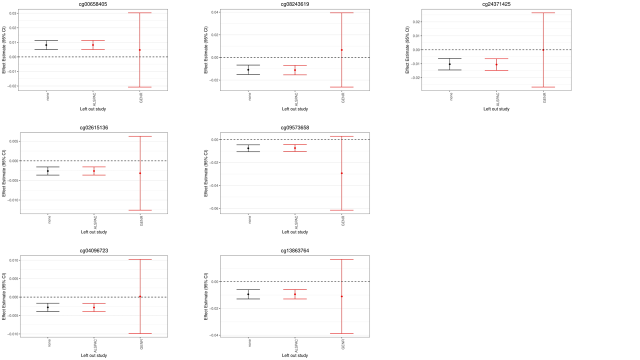
**

**Supplementary Figures 3.1-3.7. Forest plots of 7 prioritized CpGs in leave-one-out analyses: newborn meta-analysis.**

**
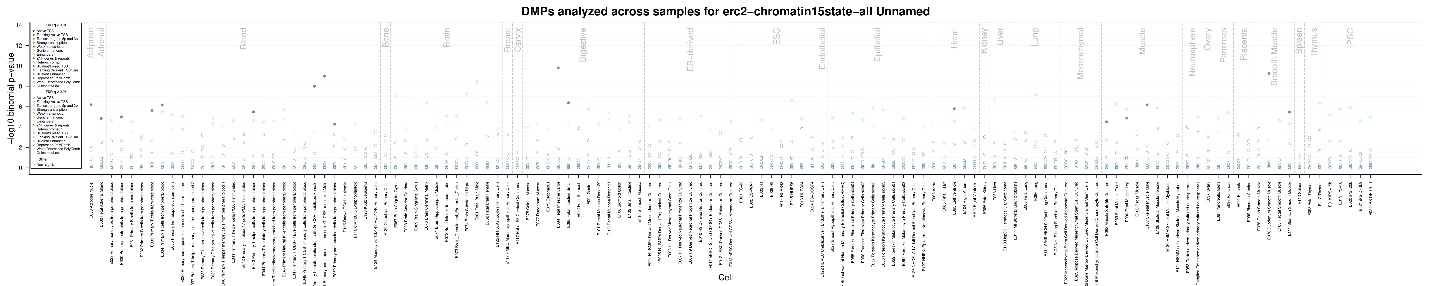
**

**Supplementary Figures 4.1-4.2. Enrichment of 109 maternal prioritized CpGs for chromatin states (upper panel) and histone marks (lower panel)**

**
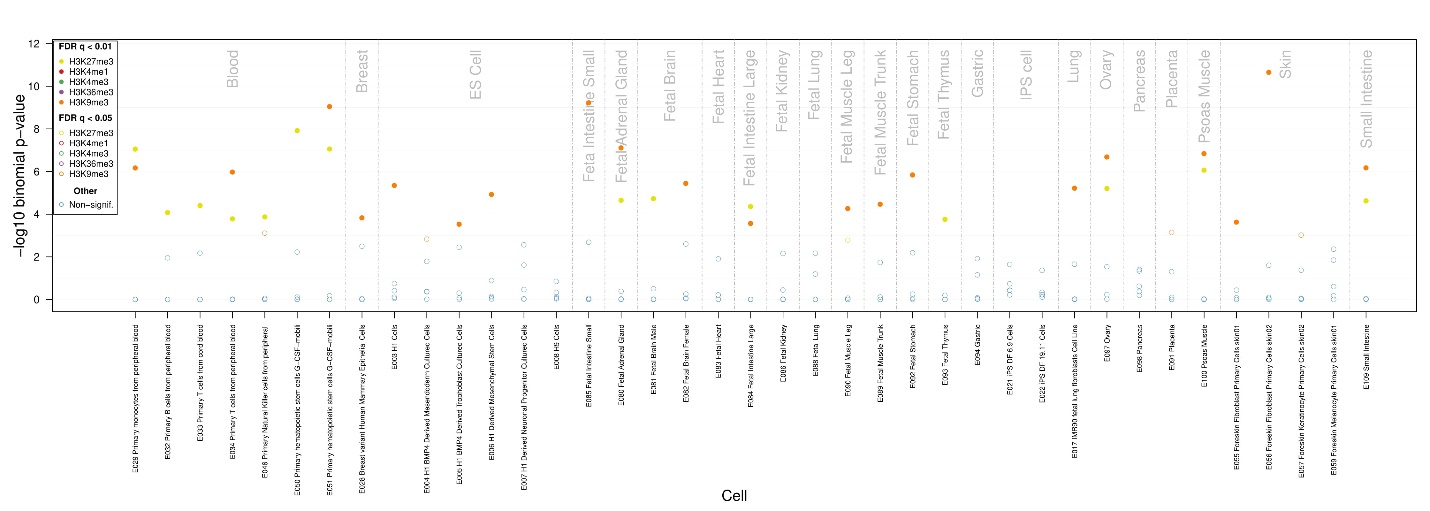
**

**
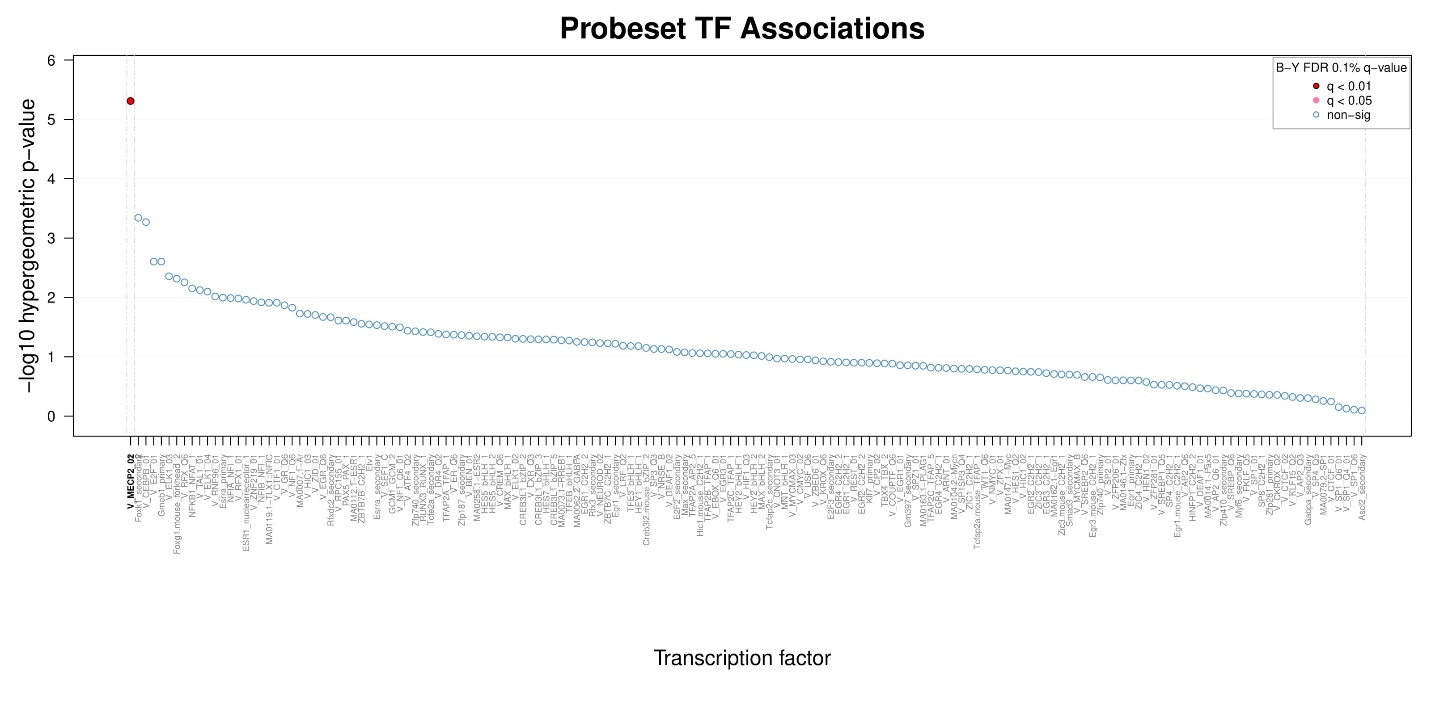
**

**
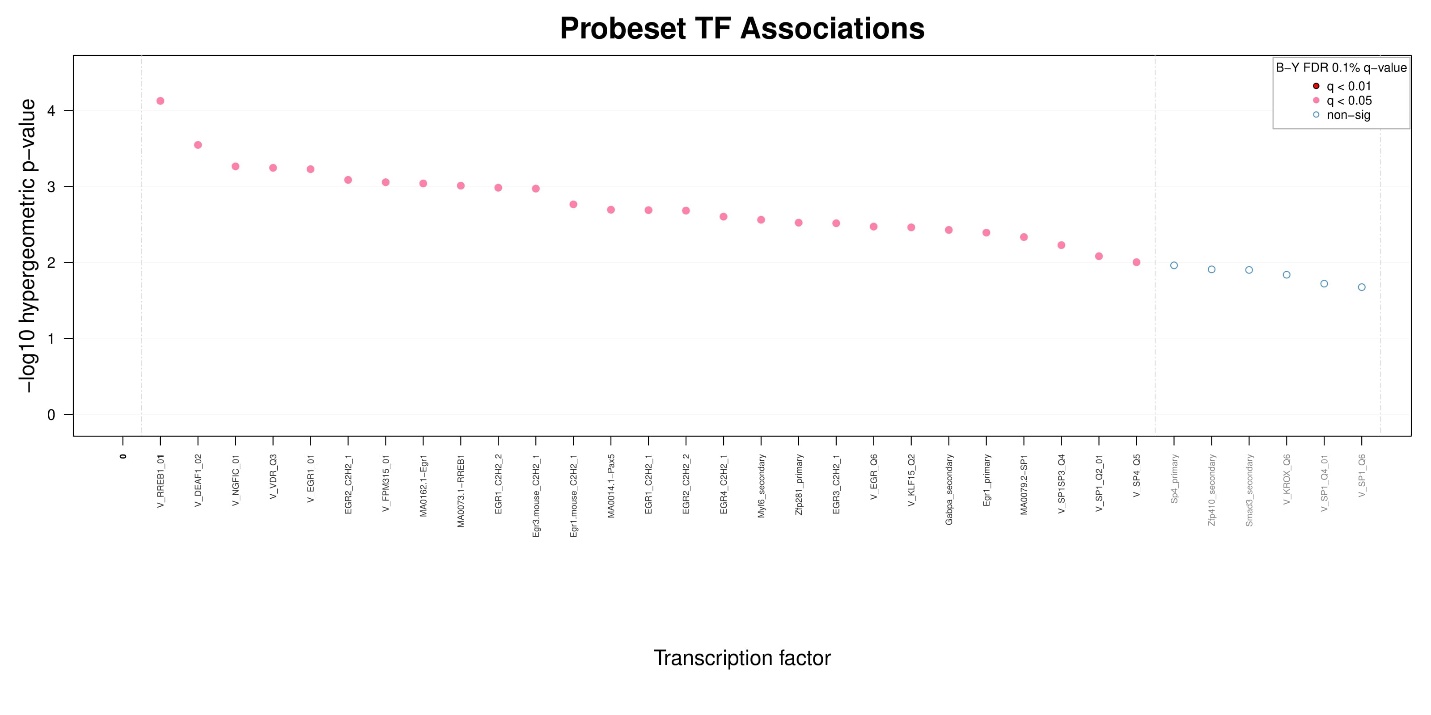
**

**Supplementary Figures 5.1-5.2. Enrichment of 109 maternal prioritized CpGs (upper panel) and 7 newborn prioritized CpGs (lower panel) for transcription factor motifs**

**SUPPLEMENTARY METHODS**

**Avon Longitudinal Study of Parents and Children (ALSPAC)**

Design and study population

The Avon Longitudinal Study of Parents and Children (ALSPAC) is a prospective pregnancy cohort study, which enrolled 14,541 pregnant women residing in Avon, United Kingdom who had expected delivery dates between April 1st, 1991 and December 31st, 1992. As described previously, detailed information has been collected on these participants and their offspring at regular intervals (1, 2). Please note that the study website contains details of all the data that is available through a fully searchable data dictionary and variable search tool: http://www.bristol.ac.uk/alspac/researchers/our-data/. As part of the Accessible Resources for Integrated Epigenomic Studies (ARIES, <http://www.ariesepigenomics.org.uk/)> project, DNA methylation was generated for 1018 mother-offspring pairs from the ALSPAC cohort. ARIES participants were selected based on availability of DNA samples at two time points for the mother (antenatal and at follow-up when the offspring were adolescents) and at three time points for the offspring (newborn, childhood (age 7), and adolescence (age 15/17)). The current study used offspring DNA methylation at all three time points. Ethical approval for the study was obtained from the ALSPAC Ethics and Law Committee and the Local Research Ethics Committees. Consent for biological samples has been collected in accordance with the Human Tissue Act (2004). Informed consent for the use of data collected via questionnaires and clinics was obtained from the participants following the recommendations of the ALSPAC Ethics and Law Committee at the time.

This study included children with information on cord blood vitamin B12 concentrations, blood DNA methylation measurements at any time point, and complete covariate data. For the meta-analysis of cord blood vitamin B12 concentrations and cord blood DNA methylation we included n=81 mother-newborn pairs. For the analyses at older ages, n=85 (early childhood) and n=83 (adolescence) children were included in the look-up analyses of cord blood vitamin B12-related differentially methylated CpGs. In total, 80 children were included in both the analysis at birth and in childhood and 78 children were included in both the analysis at birth and in adolescence.

Vitamin B12 measurements

Vitamin B12 was measured in plasma in pmol/L. Vitamin B12 concentrations were determined using a colistin sulfate-resistant strain of *Lactobacillus leichmanii* (3, 4).

DNA Methylation measurements

Methods for methylation measurements in ALSPAC have been described previously (5). Briefly, blood was collected according to standard procedures. DNA methylation assays and data pre-processing were performed at the University of Bristol as part of the ARIES project. DNA was extracted using standard protocol and was bisulfite-converted using the Zymo EZ DNA MethylationTM kit (Zymo, Irvine, CA, USA). DNA methylation was then measured using the Infinium HM450 BeadChip assay (Illumina Inc, San Diego, CA, USA), according to the standard protocol. Arrays were scanned using an Illumina iScan. An initial review of data quality was assessed using GenomeStudio (version 2011.1). A semi-random approach (sampling criteria were in place to ensure that all time points were represented on each array) was used to distribute ARIES samples across slides to minimize the possibility of potential confounding by batch. Data were normalized using the meffil R package using the functional normalization approach (6). The final DNA methylation dataset contained information on 468,828 CpGs.

Covariates

Maternal age at delivery was derived from the mother’s date of birth. Maternal socio-economic status (SES) was defined based on the UK highest qualification achieved by the mothers. The covariate is binary and CSE/none, Vocational and O-level qualifications were grouped to describe lower SES and A-level and university degree to describe higher SES. Maternal pre-pregnancy body mass index was derived from self-reported height and pre-pregnancy weight, which were collected by questionnaire during the first trimester of pregnancy. Maternal smoking during pregnancy was determined by questionnaire at the time of recruitment and defined in this study as no smoker, if mother was a never smoker or quit before second semester, or otherwise a smoker. Parity was defined as the number of previous pregnancies resulting in either a livebirth or a stillbirth. Newborn sex was obtained from obstetric records. Participants with non-white European ancestry were excluded from all analyses. We attempted to control for technical batch effects by generating surrogate variables using the SVA R package and including these directly in our models (7). Cell type proportions were estimated from the methylation data using the Salas reference set in the “FlowSorted.CordBlood.Combined.450K” Bioconductor package and the reference-based Houseman method for cord and child blood, respectively (8, 9).

**Generation R (GENR)**

Design and study population

The Generation R Study is a population-based prospective cohort study from fetal life onwards (10). Pregnant women with an expected delivery date between April 2002 and January 2006 living in the city of Rotterdam were eligible. In a subgroup of the 9,901 live-born participating children from European ancestry we measured genome-wide DNA methylation in cord blood (n=1396) and/or in venous blood at childhood age six years (n=493) and/or at age ten years (n=464). For the current analyses, we included only one child for any of up to 15 sibling pairs, based on completeness of data and if equal, randomly. The Generation R Study was approved by the medical ethical committee of Erasmus MC, University Medical Center Rotterdam and written consent was obtained for all participants.

This study included children with any measurement of vitamin B12 concentrations during pregnancy or in cord blood, blood DNA methylation measurements at any time point, and complete covariate data (n=1068). For the meta-analyses of maternal circulating vitamin B12 concentrations during pregnancy and cord blood DNA methylation, n=823 mother-newborn pairs were included in the primary model, n=803 in the folate model and n=795 in the homocysteine model. For the meta-analysis of cord blood vitamin B12 concentrations and cord blood DNA methylation we included n=948 mother-newborn pairs. In total, n=708 mother-newborn pairs had information on both maternal and cord blood vitamin B12 concentrations. For the analyses at older ages, n=284 (early childhood) and n=267 (late childhood) children were included in the look-up analyses of maternal vitamin B12-related differentially methylated CpGs. Further, n=332 (early childhood) and n=321 (late childhood) children were included in the look-up analyses of cord blood vitamin B12-related differentially methylated CpGs. For the analyses of maternal vitamin B12 concentrations during pregnancy, all children included in the analyses in childhood were also included in the analysis at birth. For the analyses of newborn vitamin B12 concentrations, n=332 children were also included in the early childhood analysis and n=300 children were also included in the late childhood analysis.

Vitamin B12 measurements

Vitamin B12 was measured in serum in pmol/L. As described in detail previously, maternal venous blood samples were drawn in early pregnancy in a dedicated research center under non-fasting conditions and cord blood samples were taken immediately after delivery (11). After collection, blood samples were stored at room temperature for a maximum of 3 hours, before being transported to the regional laboratory for processing and storage at -80°C. Vitamin B12 concentrations were analyzed in the Department of Clinical Chemistry at Erasmus MC, University Medical Centre Rotterdam. After thawing, vitamin B12 concentrations were analyzed using an immune-electro-chemo-luminescence assay on the Architect System. Concentrations below or above the analytic ranges of this assay for vitamin B12 (44-1476 pmol/l) could not be quantified. As a consequence, these concentrations were recorded as either the lower or the upper limit of the assay (maternal vitamin B12: n=1 (excluded because >5SD of population mean), cord blood vitamin B12: n=2).

DNA Methylation measurements

We used the salting-out method to extract DNA from cord or venous blood samples. Five-hundred nanograms of DNA were bisulfite converted using the EZ-96 DNA Methylation kit (Shallow) (Zymo Research Corporation, Irvine, USA). Samples were processed with the Illumina Infinium HumanMethylation450 BeadChip (Illumina Inc., San Diego, USA). Quality control and normalization were performed using the CPACOR workflow (12). Probes with a detection *P*-value ≥1E-16 were set to missing. Intensity values were quantile normalized. Arrays with technical problems, a call rate ≤95%, or a mismatch between sex of the proband and sex determined by chromosome X and Y probe intensities were removed. Probes on the sex chromosomes were removed before the analyses. We used untransformed beta-values as measures of DNA methylation. The final DNA methylation dataset contained information on 458,563 CpGs.

Covariates

As described previously, information on maternal covariates was obtained from questionnaires sent out at enrollment (10). For the current analysis, we calculated maternal age at conception from maternal age at enrollment. We categorized maternal educational level into lower (primary education), middle (secondary education) and higher (more than secondary education). For the look-up analyses in childhood, the lowest two categories were combined as only five (early childhood) and seven mothers (late childhood) had completed lower education. Pre-pregnancy body mass index was calculated from height at enrollment and self-reported pre-pregnancy weight. Smoking during pregnancy was dichotomized into no smoking or quit before second trimester versus sustained smoking. Parity was dichotomized into nulliparous or multiparous. Information on child sex was obtained from midwife and hospital records. We adjusted for batch effects by adding plate number as a covariate. For the meta-analyses, n=10 (maternal vitamin B12) and n=8 (newborn vitamin B12) participants from three batches were excluded, because these batches included four or less participants. All participants were of European ancestry. Gestational age at blood sampling was primarily estimated using the first day of the last menstrual period. If this information was unknown, or in case of an irregular menstrual cycle, gestational age was established by first trimester fetal ultrasound examination. Folate and homocysteine concentrations were measured in EDTA plasma and analyzed as described previously (11). Concentrations below or above the analytic ranges of the assay for folate (1.8-45.3 nmol/l) and homocysteine (1-50 µmol/l) could not be quantified. As a consequence, these concentrations were recorded as either the lower or the upper limit of the assay. Cell type proportions were estimated from the methylation data using the Salas reference set in the “FlowSorted.CordBlood.Combined.450K” Bioconductor package and the reference-based Houseman method for cord and child blood, respectively (8,9).

**INfancia y Medio Ambiente** **(INMA) - Sabadell**

Design and study population

The present study used data from participants recruited between 2003 and 2008 in the *de novo* cohort sited in Sabadell of the INfancia y Medio Ambiente (INMA) Project, a population-based mother–child cohort study in Spain (13). The current project uses data from European ancestry children from the Sabadell subcohort. Study website: <http://www.proyectoinma.org/>. The study has been approved by Ethical Committee of each participating center and written consent was obtained from participating parents.

This study included children with maternal vitamin B12 concentrations during pregnancy, blood DNA methylation measurements at any time point, and complete covariate data. For the meta-analysis of maternal vitamin B12 concentrations during pregnancy and DNA methylation we included n=372 mother-newborn pairs in the primary model and n=369 mother-newborn pairs in the folate model. For the analyses at older ages, n=195 (early childhood, 4 year) and n=215 (late childhood, 9 year) children were included in the look-up analyses of maternal vitamin B12-related differentially methylated CpGs. In total, up to n=114 children were included in both the analysis at birth and an analysis in childhood.

Vitamin B12 measurements

Vitamin B12 was measured in serum in pg/mL. Vitamin B12 concentrations were measured in samples collected at the end of the first trimester of pregnancy (Normative Public Health Laboratory of Bilbao, Basque Country) using a time-resolved fluoroimmunossay (AutoDelfia-PerkinElmer Life and Analytical Sciences, Wallac Oy, Turku, Finland).

DNA Methylation measurements

INMA 450k data was produced within the MEDALL, BREATHE and HELIX projects. Cord or peripheral blood was extracted using the Chemagen kit (Perkin Elmer). DNA concentration was determined by NanoDrop spectrophotometer (Thermo Scientific) and with the Quant-iT PicoGreen dsDNA Assay Kit (Life Technologies). Methylation data was produced in 3 different laboratories: in the Genome Analysis Facility of the University Medical Center Groningen (UMCG) in the Netherlands (MEDALL 0y and 4y), and in the Bellvitge Biomedical Research Institute (IDIBELL, Barcelona) (BREATHE 0y), and in the Spanish National Genotyping Center (CEGEN, Madrid) (HELIX 9y). All laboratories used the recommended Illumina protocol for the Infinium HumanMethylation450 beadchip. Briefly, 500 ng of DNA was bisulfite-converted using the EZ 96-DNA methylation kit following the manufacturer’s standard protocol, and DNA methylation measured using the Illumina Infinium HumanMethylation450 beadchip. The quality control of the methylation data obtained at birth and at 4y was done together, while the quality control of the methylation data at age 9y was done together with other samples from the HELIX project.

Cord and 4y DNA methylation data (MEDALL and BREATHE) were quality controlled and preprocessed using the minfi package (14). A series of steps were completed for quality control and data analysis. The first step was low quality sample removal. First, 2 samples with bad overall quality or with low detection P-value according to the output of the MethylAid package were removed (15). Then, we removed 3 samples whose sex was wrongly predicted using shinyMethyl (16). Following guidelines of Lehne work, we increased the stringency of the detection P-value threshold to 10E-16 and we filtered 18 samples with a call rate lower than 98% (12). The next step was normalizing data with the functional normalization method with Noob background subtraction and dye-bias correction (17). Correlation between SNPs in replicates was checked and probes not measuring SNPs were discarded. Probes with a call rate lower than 95% were also removed. Probes in the sexual chromosomes, cross-hybridizing or containing SNPs were flagged but not removed at this point. ComBat was applied to remove laboratory batch effect (18). Finally, duplicated samples were removed, prioritizing MeDALL samples over BREATHE samples. The final DNA methylation dataset contained information on 476,946 probes.

DNA methylation data at the age of 9 years (HELIX) was pre-processed using minfi R package (14). Probes not reaching a 98% call rate based on a detection P-value threshold <1E-16 were excluded (12). Samples with a call rate <98% were removed from the study. Then, data was normalized with the functional normalization method with Noob background subtraction and dye-bias correction (17). Then, we checked sex consistency using the shinyMethyl R package, and genetic consistency was checked making use of the genotype probes included in the Infinium HumanMethylation450K BeadChip and the genome-wide genotyping data when available. Batch effect (slide) was corrected using the ComBat R package (16, 18). Control probes, probes in sexual chromosomes, probes designed to detect Single Nucleotide Polymorphisms (SNPs) and probes to measure methylation levels at non-CpG sites were excluded, giving a final number of 476,946 probes.

Covariates

Information on maternal age was collected by questionnaire at enrolment (week 12 of pregnancy). Occupational social class, as a proxy for socio-economic status, was coded from the longest-held job during the pregnancy or, if the mother did not work in this time period, the last job prior to pregnancy. A few of the mothers had never worked and in these cases the father’s last job was used. Occupations were coded using the four-digit Spanish National Classification of Occupations (Clasificación Nacional de Ocupaciones, CNO94), which is closely related to the international ISCO88 coding system. Mothers were classified as "lower", "middle" or "higher". Maternal height was measured, and maternal pre-pregnancy weight was reported by the mother during the first prenatal visit. From these variables maternal pre-pregnancy body mass index was calculated. Pregnant women were asked about maternal smoking at week 12 and 32 of pregnancy. The variable was divided in two categories; no smoking or smoking at 1rst trimester only, and sustained smoking. Parity was asked through questionnaires and categorized as nulliparous vs multiparous. Child sex was abstracted from clinical records. Gestational age at blood sampling was calculated based on last menstrual period (LMP) reported at recruitment and confirmed using estimates based on ultrasound examination in the 12th week of gestation. When the difference between the LMP reported at recruitment and estimated from the ultrasound was ≥ 7 days (n=91; 16%), we estimated LMP using a quadratic regression formula (19). Folate concentrations were measured in serum. No information on maternal homocysteine concentrations during pregnancy was available. Cell type proportions were estimated from the methylation data using the Salas reference set in the “FlowSorted.CordBlood.Combined.450K” Bioconductor package and the reference-based Houseman method for cord and child blood, respectively (8, 9).

**Markers of Autism Risk Learning Early Signs (MARBLES)**

Design and study population

Markers of Autism Risk Learning Early Signs (MARBLES) is an enriched risk prospective pregnancy cohort to study autism etiology (20). This ongoing longitudinal study recruited mothers of confirmed Autism Spectrum Disorder children who were in a subsequent pregnancy or were trying to become pregnant. At the time of this analysis, there were 389 enrolled mothers that gave birth to 425 subsequent siblings between December 1, 2006 and July 1, 2016. Biological samples were collected during pregnancy, at birth, and during development. For families where multiple younger siblings were enrolled, one sibling was randomly for inclusion (where each had equal completeness of data). The MARBLES protocol was reviewed and approved by the Human Subjects Institutional Review Board (IRB) from University of California Davis.

This study included n=48 mother-newborns pairs with information on maternal vitamin B12 concentrations during pregnancy, cord blood DNA methylation measurements, and complete covariate data. It was used for a look-up analysis of the maternal vitamin B12-related differentially methylated CpGs (primary model).

Vitamin B12 measurements

Vitamin B12 was measured in serum in pg/ml in samples collected during pregnancy. When mothers had multiple measures, the one earliest in pregnancy was chosen. Vitamin B12 was measured using automated chemiluminescence in the CLIA-approved Medicine Clinical Laboratories at UC Davis Medical Center (inter-assay CV = 6.2%). The upper measurement limit for vitamin B12 is 2000 pg/ml and the upper limit for folate is 20 pg/ml, which no samples were above.

DNA Methylation measurements

At the delivery hospital, cord blood samples are collected and immediately processed and stored at -80 degrees Celsius in the UC Davis repository. After storage, cord blood samples were transferred to the Johns Hopkins Biological Repository (JHBR) laboratory where cord blood DNA was extracted from a subsample of 279 participants with 36-month diagnostic assessment data available using the DNA Midi kit (Qiagen, Valencia, CA). Samples were bisulfite treated and cleaned using the EZ DNA methylation gold kit (Zymo Research, Irvine, CA). DNA was plated randomly and assayed on the Infinium HumanMethylationEPIC BeadChip (Illumina, San Diego, CA, USA) at the Johns Hopkins SNP Center, a shared lab and informatics operation with the Center for Inherited Disease Research (Johns Hopkins University, USA). DNA methylation control gradients and between-plate repeated tissue controls were used. Samples that have mismatched predicted sex were dropped (n=3), as were samples with gestational age methylation clocks that were highly discrepant (difference with clock age > 3 weeks, n=32). There were 244 cord blood samples that passed DNA methylation quality control. Probes were dropped (n=4633) if they had detection-p (p>0.01) failure in greater than 5% of samples. Cross reactive probes (n=42967) were also dropped (21). We used the minfi library (version 1.30.0) in R (version 3.6) to process raw Illumina image files into noob background corrected methylation values (14, 22). 3IQR trimming of outlier beta values was done according to meta-analysis specifications. For the look-up analysis, only information on the 109 prioritized CpGs of the maternal primary meta-analysis (FDR-corrected P-value<0.05 and I^2^ <50%) was included in the final DNA methylation dataset.

Covariates

Demographics, maternal behaviors, food frequency and medical history were all collected via questionnaire. Maternal body mass index was calculated from pre-pregnancy weight and height that was extracted from medical records, state birth files, or self-report questionnaires where available. Maternal age at child birth was used in the current analysis. Maternal education was categorized into 3 categories: “no college degree”, “bachelor degree” and “graduate education”. Of the included mother-newborn pairs, only one mother had sustained smoking during pregnancy. We excluded this one mother and did not include maternal smoking as a covariate in models. Due to cohort design all mothers were multiparous, and thus we dropped the parity covariate from models. We adjusted for batch effects by adding plate number as a covariate. Sample size was too small to run in ethnic groups separately. We therefore included everyone together and used ancestry principal components from GWAS data as adjustment variables. Cell type proportions were estimated from the methylation data using the Salas reference set in the “FlowSorted.CordBlood.Combined.450K” Bioconductor package for cord blood (8).

**Norwegian Mother, Father and Child Cohort Study (MoBa): MoBa1 and MoBa2**

Design and study population

For MoBa1, mother-offspring pairs were selected from a substudy within the Norwegian Mother, Father and Child Cohort Study (MoBa) (23-25). The substudy was a cohort random sample plus asthma cases at age three years (26). Offspring in this study were born in 2002 to 2004. A non-overlapping subset was selected from MoBa (23-25). MoBa2 included a cohort random sample and asthma cases at age seven years and non-asthmatic controls (27). Offspring in this subset were born in 2000 to 2005. MoBa1 and MoBa2 were approved by the Regional Committee for Ethics in Medical Research in Norway and the Institutional Review Board of the National Institute of Environmental Health Sciences in the USA. Previous MoBa1 and MoBa2 publications were based on data release version 5. These analyses were analyzed using data release version 12.

This study included children with maternal vitamin B12 concentrations during pregnancy, blood DNA methylation measurements at birth, and complete covariate data. For the meta-analysis of maternal vitamin B12 concentrations during pregnancy and DNA methylation we included n=1007 (MoBa1) and n=218 (MoBa2) mother-newborn pairs in the primary model, folate model and homocysteine model. Each dataset was analyzed separately.

Vitamin B12 measurements

Vitamin B12 was measured in plasma in pmol/L. Maternal blood samples were drawn during pregnancy (median weeks gestation=18 weeks, 25th-75th percentile=16-21 weeks) in EDTA lined tubes, centrifuged within 30 minutes after collection, and stored at 4°C in the hospital where they were collected. Samples were then shipped overnight to the Biobank of MoBa at the Norwegian Institute of Public Health in Oslo. Upon receipt (1-2 days after blood collection), plasma was aliquoted onto polypropylene microtiter plates, sealed with heat-sealing foil sheets, and stored at -80°C. Plasma vitamin B12 concentrations were measured at Bevital AS (www.bevital.no) by microbiological assay, determined by a Lactobacillus leichmannii microbiological assay (28).

DNA Methylation measurements

Methylation was measured in MoBa1 first (29). The same laboratory measured DNA methylation in MoBa2 later (30). Details of how DNA methylation were measured and the quality control procedure for MoBa1 and MoBa2 have been previously described (30). The same procedures were applied to both studies. Briefly, samples of umbilical cord blood were collected at birth and stored at -80°C. DNA was bisulfite converted using the EZ-96 DNA Methylation kit (Zymo Research Corporation, Irvine, CA, USA). DNA methylation was assessed at 485,577 CpGs using Illumina’s Infinium HumanMethylation450 BeadChip (31). We used the *minfi* package in R to read the .idat files and calculate the beta methylation values at each CpG:

$$\beta=\frac{intensityofthemethylatedallele(M)}{intensityoftheunmethylatedallele(U)+intensityofthemethylatedallele(M)+100}$$

Quality control procedures were applied on the beta methylation values. We excluded 65 control probes, probes on the X chromosome (# CpGs =11,230) and probes on the Y chromosome (# CpGs =416). CpGs missing >10% of methylation values were removed (20 CpGs in MoBa1, 0 CpGs in MoBa2). We excluded samples identified by Illumina to have failed or those with an average detection P-value <0.05 across all probes (49 in MoBa1, 35 in MoBa2), as well as samples with sex mismatches (13 in MoBa1, 8 in MoBa2). We used the beta mixture quantile (BMIQ) to normalize the data (32). Extreme beta methylation values (defined as greater or less than three times the interquartile range below the 25^th^ percentile or above the 75^th^ percentile) were set to missing. A total of 1,068 samples passed quality control in MoBa1 and 685 samples passed quality control in MoBa2. In data release version 12, eleven participants dropped out of MoBa1 and 0 dropped out of MoBa2. Genotype data were measured using Illumina HumanCore. We ran principal components to identify and exclude samples that were ancestry outliers (6in MoBa1, 4 in MoBa2). The final DNA methylation dataset contained information on 473,844 (MoBa1) and 473,748 (MoBa2) probes. DNA methylation was not measured at older ages.

Covariates

MoBa participants completed the first questionnaire around the 17^th^ week of gestation and self-reported their age, education, pre-pregnancy weight, and height. MoBa did not collect information on maternal age at conception, so we used maternal age at the first questionnaire. Maternal education was defined as: 1) less than high school or completed high school, 2) some college, 3) college or higher. Maternal pre-pregnancy body mass index was calculated from self-reported weight and height. Maternal smoking status was determined based on measured cotinine levels around the 18^th^ week of gestation and self-reported smoking information at collected at the 17^th^ week of gestation, 30^rd^ week of gestation, and 6^th^ month after birth. MoBa participants were linked to the Medical Birth Registry of Norway to collect information on infant sex. We used ComBat from the sva package in R for batch correction (18). We also adjusted for asthma status at 3 years old in MoBa1 as a selection factor. Information on individual gestational age at blood sampling during pregnancy was not available for the current analysis and thus not included as a covariate in the models. All included children were of European ancestry based on principal components from genomic data. Plasma levels of total homocysteine (tHcy) were assayed using a gas chromatography-mass spectrometry method based on methylchloroformate derivatization (33). Plasma folate concentration was measured using a chloramphenicol-resistant strain of Lactobacillus casei, which measures biologically active folate species, predominantly 5-methyl-tetrahydrofolate (34) The CV for this assay corresponds to 4% within-day and 5% between days, at population median (27). Cell type proportions were estimated from the methylation data using the Salas reference set in the “FlowSorted.CordBlood.Combined.450K” Bioconductor package for cord blood (8).

**ACKNOWLEDGEMENTS**

**Avon Longitudinal Study of Parents and Children (ALSPAC)**

We are extremely grateful to all the families who took part in this study, the midwives for their help in recruiting them, and the whole ALSPAC team, which includes interviewers, computer and laboratory technicians, clerical workers, research scientists, volunteers, managers, receptionists, and nurses. Please note that the ALSPAC study website (http://www.bristol.ac.uk/alspac/researchers/our-data/) contains details of all the data that is available through a fully searchable data dictionary and variable search tool. We would like to acknowledge Tom Gaunt, Oliver Lyttleton, Sue Ring, Nabila Kazmi, and Geoff Woodward for their earlier contribution to the generation of ARIES data (ALSPAC methylation data).

**Generation R (GENR)**

The Generation R Study is conducted by Erasmus MC, University Medical Center Rotterdam in close collaboration with the School of Law and Faculty of Social Sciences of the Erasmus University Rotterdam, the Municipal Health Service Rotterdam area, Rotterdam, the Rotterdam Homecare Foundation, Rotterdam and the Stichting Trombosedienst & Artsenlaboratorium Rijnmond (STAR-MDC), Rotterdam. We gratefully acknowledge the contribution of children and parents, general practitioners, hospitals, midwives and pharmacies in Rotterdam. The study protocol was approved by the Medical Ethical Committee of Erasmus MC. Written informed consent was obtained for all participants. The generation and management of the Illumina 450K methylation array data (EWAS data) for the Generation R Study was executed by the Human Genotyping Facility of the Genetic Laboratory of the Department of Internal Medicine, Erasmus MC, and the Netherlands. We thank Mr. Michael Verbiest, Ms. Mila Jhamai, Ms. Sarah Higgins, Mr. Marijn Verkerk and Dr. Lisette Stolk for their help in creating the EWAS database. We thank Dr. A.Teumer for his work on the quality control and normalization scripts.

**INfancia y Medio Ambiente** **(INMA) - Sabadell**

The authors would particularly like to thank all the participants for their generous collaboration. A full roster of the INMA Project Investigators can be found at <http://www.proyectoinma.org/presentacion-inma/listado-investigadores/en_listado-investigadores.html>.

**Markers of Autism Risk Learning Early Signs (MARBLES)**

We are grateful to the participants of the MARBLES Study, without whom this work would not have been possible.

**Norwegian Mother, Father and Child Cohort Study (MoBa): MoBa1 and MoBa2**

We are grateful to all the participating families in Norway who took part in this on-going cohort study. We thank Jakob Mjånes and Elin Alsaker of the National Institute of Public Health (Bergen, Norway), Dr. Frank Day of NIEHS and Dr. Jianping Jin of Westat (Durham, NC) for expertise in data management and computational assistance.

**FUNDING**

**Avon Longitudinal Study of Parents and Children (ALSPAC)**

GM and GCS are members of the MRC Integrative Epidemiology Unit, which receives funds from the University of Bristol and United Kingdom Medical Research Council [MC_UU_00011/1, MC_UU_00011/5 and MC_UU_00011/6]. GCS’s contribution to this work is supported by the Medical Research Council [New Investigator Research Grant, MR/S009310/1]. GM, LJ and GCS’s contributions are supported by the European Joint Programming Initiative “A Healthy Diet for a Healthy Life” (JPI HDHL, NutriPROGRAM project, UK MRC MR/S036520/1]. The UK Medical Research Council and the Wellcome Trust (Grant ref: 217065/Z/19/Z) and the University of Bristol provide core support for ALSPAC. A comprehensive list of grants funding is available on the ALSPAC website (http://www.bristol.ac.uk/alspac/external/documents/grant-acknowledgements.pdf). The Accessible Resource for Integrated Epigenomics Studies (ARIES) which generated large scale methylation data was funded by the UK Biotechnology and Biological Sciences Research Council (BB/I025751/1 and BB/I025263/1). Additional epigenetic profiling on the ALSPAC cohort was supported by the UK Medical Research Council Integrative Epidemiology Unit and the University of Bristol (MC_UU_12013_1, MC_UU_12013_2, MC_UU_12013_5 and MC_UU_12013_8), the United States National Institute of Health (5RO1AI121226-02) and National Institute of Child and Human Development grant (R01HD068437). The funders had no role in study design, data collection and analysis, decision to publish, or preparation of the manuscript. This publication is the work of the authors and Gemma Sharp will serve as guarantors for the contents of this paper. The views expressed in this paper are those of the authors and not necessarily any funders. The funders had no influence on the content of the paper.

**Generation R (GENR)**

The general design of the Generation R Study is made possible by ﬁnancial support from the Erasmus MC, University Medical Centre Rotterdam, Erasmus University Rotterdam, the Netherlands Organization for Health Research and Development (ZonMw), the Netherlands Organization for Scientific Research (NWO), the Ministry of Health, Welfare and Sport and the Ministry of Youth and Families. The EWAS data was funded by a grant to VWVJ from the Netherlands Genomics Initiative (NGI)/Netherlands Organization for Scientific Research (NWO) Netherlands Consortium for Healthy Aging (NCHA; project number 050-060-810), by funds from the Genetic Laboratory of the Department of Internal Medicine, Erasmus MC, and by a grant from the National Institute of Child and Human Development (R01HD068437). VWVJ received funding from the European Research Council (ERC-2014-CoG-648916). The project was supported by funding from the European Union's Horizon 2020 research and innovation program under grant agreements No 733206 (LifeCycle), 874739 (LongITools) and 824989 (EUCAN-Connect), and from the European Joint Programming Initiative “A Healthy Diet for a Healthy Life” (JPI HDHL, NutriPROGRAM project, ZonMw the Netherlands no.529051022 and PREcisE project ZonMw the Netherlands no.529051023).

**INfancia y Medio Ambiente** **(INMA) - Sabadell**

This study was funded by grants from Instituto de Salud Carlos III (Red INMA G03/176; CB06/02/0041; PI041436), PI081151 incl. FEDER funds), Generalitat de Catalunya-CIRIT 1999SGR 00241, Fundació La marató de TV3 (090430), the European Community’s Seventh Framework Programme under grant agreements no 308333 (HELIX project) and 261357 (MEDALL project), the European Community’s Seventh Framework Programme - European Research Council (ERC) grant agreement no 268479 (BREATHE Project), the European Union’s Horizon 2020 research and innovation programme under grant agreement no 733206 (LIFECYCLE), and the European Joint Programming Initiative “A Healthy Diet for a Healthy Life” (JPI HDHL and Instituto de Salud Carlos III) under the grant agreement no AC18/00006 (NutriPROGRAM project).

We acknowledge support from the Spanish Ministry of Science and Innovation through the “Centro de Excelencia Severo Ochoa 2019-2023” Program [CEX2018-000806-S], and support from the Generalitat de Catalunya through the CERCA Program.

**Markers of Autism Risk Learning Early Signs (MARBLES)**

The MARBLES study and this work has been supported by a grant from the Allen Foundation, pilot funding from the MIND Institute, EPA STAR grant #RD-83329201, and NIH grants: R24ES028533, R01ES028089, R01ES020392, R01ES025574, P01ES011269, and K12HD051958. These supporting organizations had no role in the design and conduct of the work; collection, management, analysis, and interpretation of the data; preparation, review, or approval of the manuscript; and decision to submit the manuscript for publication. The findings and conclusions in this report are those of the authors and do not necessarily represent the official position of the National Institutes of Health or EPA.

**Norwegian Mother, Father and Child Cohort Study (MoBa): MoBa1 and MoBa2**

The Norwegian Mother and Child Cohort Study are supported by the Norwegian Ministry of Health and Care Services and the Ministry of Education and Research, NIH/NIEHS (contract no N01-ES-75558), NIH/NINDS (grant no.1 UO1 NS 047537-01 and grant no.2 UO1 NS 047537-06A1). For this work, MoBa 1 and 2 were supported by the Intramural Research Program of the NIH, National Institute of Environmental Health Sciences (Z01-ES-49019) and the Norwegian Research Council/BIOBANK (grant no 221097). This work was partly supported by the Research Council of Norway through its Centres of Excellence funding scheme, project number 262700.

**REFERENCES**

1. Boyd A, Golding J, Macleod J, Lawlor DA, Fraser A, Henderson J, et al. Cohort Profile: the 'children of the 90s'--the index offspring of the Avon Longitudinal Study of Parents and Children. Int J Epidemiol. 2013;42(1):111-27.

2. Fraser A, Macdonald-Wallis C, Tilling K, Boyd A, Golding J, Davey Smith G, et al. Cohort profile: the Avon Longitudinal Study of Parents and Children: ALSPAC mothers cohort. Int J Epidemiol. 2013;42(1):97-110.

3. Kelleher BP, Walshe KG, Scott JM, OBroin SD. Microbiological assay for vitamin B12 with use of a colistin-sulfate-resistant organism. Clin Chem. 1987;33:52-4.

4. Refsum H, Johnston C, Guttormsen AB, Nexo E. Holotranscobalamin and total transcobalamin in human plasma: determination, determinants, and reference values in healthy adults. Clin Chem. 2006;52:129-37.

5. Relton CL, Gaunt T, McArdle W, Ho K, Duggirala A, Shihab H, et al. Data Resource Profile: Accessible Resource for Integrated Epigenomic Studies (ARIES). Int J Epidemiol. 2015;44(4):1181-90.

6. Min J, Hemani G, Davey Smith G, Relton C, Suderman M. Meffil: efficient normalization and analysis of very large DNA methylation datasets. Bioinformatics. 2018;1:34(23):3983-9.

7. Leek JT, Storey JD. Capturing heterogeneity in gene expression studies by ‘surrogate variable analysis’. PLoS Genetics. 2007;3:e161.

8. Gervin K, Salas LA, Bakulski KM, van Zelm MC, Koestler DC, Wiencke JK, et al. Systematic evaluation and validation of reference and library selection methods for deconvolution of cord blood DNA methylation data. Clin Epigenetics. 2019;11(1):125.

9. Houseman EA, Molitor J, Marsit CJ. Reference-free cell mixture adjustments in analysis of DNA methylation data. Bioinformatics. 2014;30(10):1431-9.

10. Kooijman MN, Kruithof CJ, van Duijn CM, Duijts L, Franco OH, van IJzendoorn MH, et al. The Generation R Study: design and cohort update 2017. Eur J Epidemiol. 2016;31(12):1243-64.

11. Bergen NE, Jaddoe VWV, Timmermans S, Hofman A, Lindemans J, Russcher H, et al. Homocysteine and folate concentrations in early pregnancy and the risk of adverse pregnancy outcomes: the Generation R Study. BJOG. 2012;119(6):739–51.

12. Lehne B, Drong AW, Loh M, Zhang W, Scott WR, Tan ST, et al. A coherent approach for analysis of the Illumina HumanMethylation450 BeadChip improves data quality and performance in epigenome-wide association studies. Genome Biol. 2015:16:37.

13. Guxens M, Ballester F, Espada M, Fernandez MF, Grimalt JO, Ibarluzea J, et al. Cohort profile: the INMA--INfancia y Medio Ambiente--(Environment and Childhood) Project. Int J Epidemiol. 2012;41:930-40.

14. Aryee MJ, Jaffe AE, Corrada-Bravo H, Ladd-Acosta C, Feinberg AP, Hansen KD, et al. Minfi: A flexible and comprehensive Bioconductor package for the analysis of Infinium DNA Methylation microarrays. Bioinformatics. 2014;30(10):1363-9.

15. van Iterson M, Tobi EW, Slieker RC, den Hollander W, Luijk R, Slagboom PE, et al. MethylAid: visual and interactive quality control of large Illumina 450k datasets. Bioinformatics. 2014;30(23):3435–7.

16. Fortin JP, Fertig E, Hansen K. shinyMethyl: interactive quality control of Illumina 450k DNA methylation arrays in R. 2014. p. F1000Res.3:175.

17. Fortin JP, Labbe A, Lemire M, Zanke BW, Hudson TJ, Fertig EJ, et al. Functional normalization of 450k methylation array data improves replication in large cancer studies. Genome Biol. 2014;15(12):503.

18. Johnson WE, Li C, Rabinovic. A. Adjusting batch effects in microarray expression data using empirical Bayes methods. Biostatistics. 2007;8(1):118-27

19. Westerway SC, Davison A, Cowell S. Ultrasonic fetal measurements: new Australian standards for the new millennium. ANZJOG. 2000;40:297-302.

20. Hertz-Picciotto I, Schmidt RJ, Walker CK, Bennett DH, Oliver M, Shedd-Wise KM, et al. A prospective study of environmental exposures and early biomarkers in autism spectrum disorder: Design, protocols, and preliminary data from the marbles study. Environ Health Perspect. 2018;126(117004).

21. Pidsley R, Zotenko E, Peters TJ, Lawrence MG, Risbridger GP, Molloy P, et al. Critical evaluation of the Illumina MethylationEPIC BeadChip microarray for whole-genome DNA methylation profiling. Genome biology. 2016;17(1):208-.

22. Triche TJ, Weisenberger DJ, Van Den Berg D, Laird PW, Siegmund KD. Low-level processing of illumina infinium dna methylation beadarrays. Nucleic Acids Res. 2013;41:e90.

23. Magnus P, Irgens LM, Haug K, Nystad W, Skjaerven R, Stoltenberg C, et al. Cohort profile: the Norwegian Mother and Child Cohort Study (MoBa). Int J Epidemiol. 2006;35(5):1146-50.

24. Magnus P, Birke C, Vejrup K, Haugan A, Alsaker E, Daltveit AK, et al. Cohort profile update: The norwegian mother and child cohort study (moba). Int J Epidemiol. 2016;45:382-8.

25. Ronningen KS, Paltiel L, Meltzer HM, Nordhagen R, Lie KK, Hovengen R, et al. The biobank of the norwegian mother and child cohort study: A resource for the next 100 years. Eur J Epidemiol. 2006;21:619-25.

26. Haberg SE, London SJ, Nafstad P, Nilsen RM, Ueland PM, Vollset SE, et al. Maternal folate levels in pregnancy and asthma in children at age 3 years. J Allergy Clin Immunol. 2011;127(1):262-4, 4 e1.

27. Joubert BR, den Dekker HT, Felix JF, Bohlin J, Ligthart S, Beckett E, et al. Maternal plasma folate impacts differential DNA methylation in an epigenome-wide meta-analysis of newborns. Nat Commun. 2016;10(7):10577.

28. Kelleher BP, Broin SD. Microbiological assay for vitamin B12 performed in 96-well microtitre plates. J Clin Pathol. 1991;44(7):592-5.

29. Joubert BR, Haberg SE, Nilsen R.M., Wang X, Vollset SE, Murphy SK, et al. 450K epigenome-wide scan identifies differential DNA methylation in newborns related to maternal smoking during pregnancy. Environ Health Perspect. 2012;120(10):1425-31.

30. Joubert BR, Felix JF, Yousefi P, Bakulski KM, Just AC, Breton C, et al. DNA Methylation in Newborns and Maternal Smoking in Pregnancy: Genome-wide Consortium Meta-analysis. Am J Hum Genet. 2016;98(4):680-96.

31. Bibikova M, Barnes B, Tsan C, Ho V, Klotzle B, Le JM, et al. High density DNA methylation array with single CpG site resolution. Genomics. 2011;98(4):288-95.

32. Teschendorff AE, Marabita F, Lechner M, Bartlett T, Tegner J, Gomez-Cabrero D, et al. A beta-mixture quantile normalization method for correcting probe design bias in Illumina Infinium 450 k DNA methylation data. Bioinformatics. 2013;29(2):189-96.

33. Windelberg A, Arseth O, Kvalheim G, Ueland PM. Automated assay for the determination of methylmalonic acid, total homocysteine, and related amino acids in human serum or plasma by means of methylchloroformate derivatization and gas chromatography-mass spectrometry. Clin Chem. 2005;51(11):2103-9.

34. O'Broin S, Kelleher B. Microbiological assay on microtitre plates of folate in serum and red cells. J Clin Pathol. 1992;45(4):344-7.
